# Defining rules governing recognition and Fc-mediated effector functions to the HIV-1 co-receptor binding site

**DOI:** 10.1186/s12915-020-00819-y

**Published:** 2020-07-21

**Authors:** William D. Tolbert, Rebekah Sherburn, Neelakshi Gohain, Shilei Ding, Robin Flinko, Chiara Orlandi, Krishanu Ray, Andrés Finzi, George K. Lewis, Marzena Pazgier

**Affiliations:** 1 Infectious Disease Division, Department of Medicine, Uniformed Services University of the Health Sciences, 4301 Jones Bridge Road, Bethesda, MD 20814-4712 USA; 2 Division of Vaccine Research of Institute of Human Virology, University of Maryland School of Medicine, Baltimore, USA; 3 Centre de Recherche du CHUM, Université de Montréal, Montreal, Quebec Canada; 4 Département de Microbiologie, Infectiologie et Immunologie, Université de Montréal, Montreal, Quebec Canada; 5 Department of Microbiology and Immunology, McGill University, Montreal, Quebec Canada

**Keywords:** HIV-1, Env, Co-receptor binding site, ADCC, N12-i2

## Abstract

**Background:**

The binding of HIV-1 Envelope glycoproteins (Env) to host receptor CD4 exposes vulnerable conserved epitopes within the co-receptor binding site (CoRBS) which are required for the engagement of either CCR5 or CXCR4 co-receptor to allow HIV-1 entry. Antibodies against this region have been implicated in the protection against HIV acquisition in non-human primate (NHP) challenge studies and found to act synergistically with antibodies of other specificities to deliver effective Fc-mediated effector function against HIV-1-infected cells. Here, we describe the structure and function of N12-i2, an antibody isolated from an HIV-1-infected individual, and show how the unique structural features of this antibody allow for its effective Env recognition and Fc-mediated effector function.

**Results:**

N12-i2 binds within the CoRBS utilizing two adjacent sulfo-tyrosines (TYS) for binding, one of which binds to a previously unknown TYS binding pocket formed by gp120 residues of high sequence conservation among HIV-1 strains. Structural alignment with gp120 in complex with the co-receptor CCR5 indicates that the new pocket corresponds to TYS at position 15 of CCR5. In addition, structure-function analysis of N12-i2 and other CoRBS-specific antibodies indicates a link between modes of antibody binding within the CoRBS and Fc-mediated effector activities. The efficiency of antibody-dependent cellular cytotoxicity (ADCC) correlated with both the level of antibody binding and the mode of antibody attachment to the epitope region, specifically with the way the Fc region was oriented relative to the target cell surface. Antibodies with poor Fc access mediated the poorest ADCC whereas those with their Fc region readily accessible for interaction with effector cells mediated the most potent ADCC.

**Conclusion:**

Our data identify a previously unknown binding site for TYS within the assembled CoRBS of the HIV-1 virus. In addition, our combined structural-modeling-functional analyses provide new insights into mechanisms of Fc-effector function of antibodies against HIV-1, in particular, how antibody binding to Env antigen affects the efficiency of ADCC response.

## Background

The human immunodeficiency virus (HIV-1) contains only one viral glycoprotein on its surface; the envelope (Env) spike, which is responsible for both target cell recognition and virus-host fusion. The Env spike consists of a non-covalent trimeric assembly of exterior gp120 and transmembrane gp41 glycoproteins [1, 2]. During viral entry, the recognition step is mediated by the specific interaction of the exterior gp120 subunit with the primary receptor at the target cell surface, CD4 [3]. CD4 binding induces conformational changes in the trimer, leading to the formation of the gp120 bridging sheet which, together with the stem of the gp120 third variable region (V3), constitutes the Env interaction site for co-receptor binding (CoRBS), allowing either CCR5 or CXCR4 to bind [4, 5]. Together, CD4-triggering and engagement of a co-receptor enable fusion of the viral and host cell membranes. The transitional structures arising within the Env spike post-CD4 attachment in the HIV-1 entry process are referred to as “CD4-induced” (CD4i) and constitute effective targets for humoral immune responses [6–10]. These CD4i targets include highly conserved epitopes that are located within/proximal to the assembled CoRBS [11–14] and epitopes elsewhere in the protein, including the C1/C2 region of gp120 [6–8, 15–18]. The CoRBS and C1C2 epitopes are commonly referred to as Cluster C and Cluster A epitope regions, respectively [15, 19]. CD4i epitope targets, including the Cluster C and A regions, are not fully exposed on the free Env spike, and anti-Env antibodies specific for these regions show enhanced affinity for the gp120-CD4 complex as compared to un-triggered gp120 [20]. The structure of gp120 in complex with both CD4 and CCR5 has recently been solved by cryo-electron microscopy and revealed that CCR5 most likely acts to stabilize the gp120 in CD4-bound conformation and to moor the complex close to the cell surface [21].

Several lines of evidence point towards the involvement of the CD4i antibody class in protection against HIV-1 acquisition or/and post-infection control during natural infection and in vaccine settings [17, 22–24]. The CD4i Cluster A antibodies were implicated in the protective effect of the RV144 vaccine regimen in humans [25, 26] and a mix of Cluster A and CoRBS antibodies induced by the covalently constrained gp120-CD4 complex vaccine (full-length single chain (FLSC) vaccine [27]) afforded heterologous protection against SHIV162p3 and SIVmac251 in non-human primate (NHP) challenge studies [23, 28]. Interestingly, Cluster A antibodies impacted the virus solely through Fc-mediated effector mechanisms, without direct neutralizing activity and were capable of cytolytic activity through antibody-dependent cellular cytotoxicity (ADCC) [15]. In contrast, less is known about the exact mechanisms of the protective action of CoRBS antibodies. Historically, such antibodies were classified solely based on their direct neutralizing activities; not much was known about their involvement in Fc-mediated processes. Capable of direct neutralization of mostly Tier 1 viruses, in the absence of complement or effector cells, CoRBS Abs were viewed as moderately to weakly neutralizing and overall not capable of neutralizing most circulating isolates [9, 29, 30]. Furthermore, the neutralization potency in this class was shown to directly correlate with unusual antibody structural features with the most effective neutralizers having long, tyrosine-*O*-sulfated CDR H3s [31–35]. It was shown that CoRBS Abs containing tyrosines modified by post-translational *O*-sulfation were able to mimic the interaction mediated by the N-terminus of CCR5, which contains up to four *O*-sulfo-tyrosines, with a minimum of two required for HIV-1 cell entry [34, 36].

We recently performed a systematic analysis of a panel of 33 CD4i monoclonal antibodies (mAbs) directed at the CoRBS, Cluster C region, and defined their fine epitope specificities, neutralization, and ADCC potencies [15, 19]. We detected, consistent with previous reports [9, 29, 30, 32–35], a substantial neutralization diversity among antibodies directed at Cluster C epitopes, with the most potent being those that bind epitopes overlapping the classical 17b epitope and having tyrosine-*O*-sulfated CDR H3s. Most interestingly, our studies were the first to systematically rank the breadth and potency of CoRBS epitopes as functional ADCC targets. We showed that most mAbs specific for these epitopes mediated significant ADCC against target cells coated with AT-2 inactivated viral particles [15]. Interestingly, the CoRBS antibodies were also recently found to act synergistically with Cluster A antibodies to promote FcγRIIIa engagement and to mediate ADCC of CD4^+^ HIV-infected cells and HIV-infected cells pre-treated with small CD4 mimetics [19, 37, 38].

Here, we selected the mAb N12-i2, a representative antibody of the Cluster C panel [15], and described the molecular details of its interaction with the Env antigen. N12-i2, similar to other CoRBS-specific antibodies, binds within the assembled co-receptor binding site but utilizes a unique binding mode that involves two sulfoTYSs, one of which occupies a newly defined TYS binding pocket. Furthermore, in order to better understand the mechanisms governing Fc-effector mechanism to the CoRBS epitopes, we examined if the fine epitope specificity and mode of binding of N12-i2 and other CoRBS-specific antibodies contribute to the effectiveness of Env engagement and Fc-mediated effector activities in the elimination of CEM-NKR cells sensitized with HIV-1 gp120 BaL or infected with the ADA virus.

## Results

### N12-i2 targets the co-receptor binding site of HIV-1 Env

As initially assessed [15], N12-i2 recognizes the transitional epitope that is exposed on the HIV-1 Env trimer subsequent to CD4 binding. Competitive binding assays with the well-characterized CoRBS-specific mAbs 17b [9, 13] and 19e [11] placed N12-i2 within the C1 subgroup of the Cluster C region [15] as N12-i2 cross-competed for both 17b and 19e epitopes with equal potency. The previous structural and biophysical analysis of the antigen-binding fragment (Fab) of N12-i2 in the unbound state also indicated interesting structural features in its antigen-binding site with a 23-residue long CDR H3 containing two sulfoTyrs. To precisely identify the binding site of N12-i2 within Env and to describe the molecular details of how this tyrosine-sulfated antibody anchors the antigen, we determined and refined, to 3.2-Å resolution, the N12-i2 Fab in complex with the extended core of clade A/E isolate 93TH057 (gp120_93TH057_ core_e_) and the CD4 peptide mimetic M48U1 (Table 1).

**Table 1:** Data collection and refinement statistics

	Fab N12-i2-gp120_93TH057_-M48U1 (data set 1)	Fab N12-i2-gp120_93TH057_-M48U1 (data set 2)
**Data collection**		
Wavelength, Ǻ	0.9795	0.9795
Space group	P2_1_2_1_2_1_	P2_1_2_1_2_1_
Cell parameters		
*a*, *b*, c, Å	52.7, 69.5, 213.5	52.7, 69.5, 213.5
*α*, *β*, *γ*, °	90, 90, 90	90, 90, 90
Molecules/a.u.	4	4
Resolution, (Å)	50–3.2 (3.26–3.2)	50–3.2 (3.26–3.25)
# of reflections		
Total	52,155	60,052
Unique	12,129	12,777
*R*_merg_^a^, %	32.1 (52.1)	29.5 (100)
*R*_pim_^b^, %	15.6 (43.4)	14.8 (57.7)
CC_1/2_^c^	0.931 (0.611)	0.96 (0.466)
*I*/*σ*	6.2 (1.1)	7.0 (1.25)
Completeness, %	88.5 (66.9)	96.9 (97.7)
Redundancy	4.3 (1.9)	4.7 (4.6)
**Refinement statistics**		
Resolution, Å	50.0–3.2	50.0–3.25
*R*^d^, %	24.1	23.6
*R*_free_^e^, %	30.1	28.3
# of atoms		
Protein	6130	6130
Ligand	190	190
Overall *B* value (Å)^2^		
Protein	81	80
Ligand	74	71
Root mean square deviation		
Bond lengths, Å	0.003	0.004
Bond angles, °	0.7	0.8
Ramachandran^f^		
Favored, %	90.1	90.2
Allowed, %	98.5	98.3
Outliers, %	1.5	1.7
PDB ID	6W4M	–

Values in parentheses are for highest-resolution shell

^a^*R*_merge_ = ∑│*I*− < *I* > │/∑*I*, where *I* is the observed intensity and < *I* > is the average intensity obtained from multiple observations of symmetry-related reflections after rejections

^b^*R*_pim_ = as defined by Weiss [39]

^c^CC_1/2_ = as defined by Karplus and Diederichs [40]

^d^*R* = ∑║*F*_o_│ − │ *F*_c_║/∑│*F*_o_ │, where *F*_o_ and *F*_c_ are the observed and calculated structure factors, respectively

^e^*R*_free_ = defined by Brünger [41]

^f^Calculated with MolProbity

**Fig. 1:**
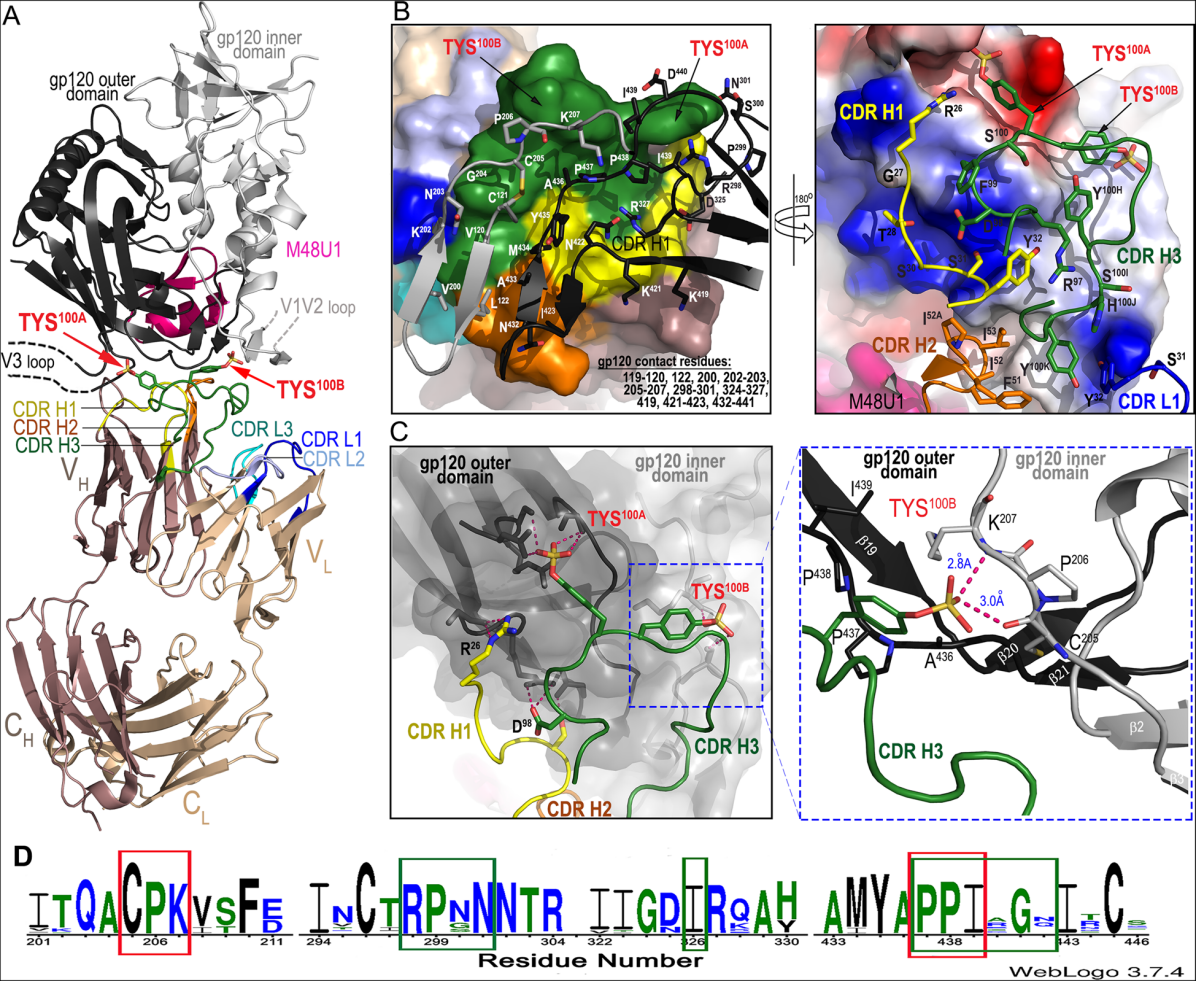
Crystal structure of N12-i2 Fab-gp120_93TH057_ core_e_-M48U1 complex. **a** A ribbon diagram of the complex. The gp120 outer and inner domains are black and gray, respectively, with the CDRs of N12-i2 colored as indicated. The two sulfoTyrs essential for N12-i2 binding, TYS^100A^ and TYS^100B^, are shown as sticks. The V1V2 and V3 loops not present in the gp120_93TH057_ core_e_ construct are shown as broken lines. **b** N12-i2 Fab-gp120_93TH057_ core_e_ interface. The Fab molecular surface colored yellow for CDR H1, orange for CDR H2, green for CDR H3, blue for CDR L1, light blue for CDR L2 and cyan for CDR L3. gp120 contact residues are shown as sticks and outer and inner domains are shown as in **a**. gp120 contact residues are listed. A 180° view shows details of Fab interaction with the gp120 surface. The gp120 molecular surface is colored based on electrostatic charge, red negative and blue positive. The N12-i2 Fab is displayed as a ribbon diagram with contact residues shown as sticks. Fab contact residues are listed. **c** Hydrogen bond network formed at the N12-i2 Fab-gp12093TH057 core_e_ interface. The molecular surface is displayed over a gp120 molecule and the gp120 outer and inner domains are shown in black and gray, respectively. The blow-up view shows details of TYS 100B interaction with gp120_93TH057_ core_e_. Residues contributing to TYS 100B binding are shown as sticks, and the network of hydrogen bonds is shown with dashed lines. See also Additional file 1: Table S1. **d** Consensus sequences for gp120 contact residues as defined by a 5-Å cutoff for N12-i2 sulfotryosines 100A (green) and 100B (red) as determined by WebLogo [42]. Approximately 32,000 aligned sequences from the HIV Sequence Database Compendium (https://www.hiv.lanl.gov/content/sequence/HIV/COMPENDIUM/compendium.html) were used as input. Insertions or deletions relative to HXBC2 were excluded for clarity and residue position given relative to HXBC2

N12-i2 binds within the CoRBS of the CD4-triggered gp120 and engages with binding residues of the bridging sheet and the base of the V3 loop of gp120 (Fig. 1). The bridging sheet is assembled within the exterior gp120 subunit upon CD4 binding to the HIV-1 Env trimer and consists of a four-stranded β-sheet formed by two strands from the outer domain and two strands from the inner domain with the latter forming the base for the variable loops 1 and 2 (V1V2 loop) [14, 43–45]. The movement of the V1V2 loop that generates the bridging sheet also exposes the V3 loop (both the crown and the stem) normally covered in the un-triggered trimer [45, 46] and forms the binding site for the co-receptor. N12-i2 engages elements of the CoRBS within the inner domain (residues: 119–120, 122 and 200, 202–203, and 205–207, buried surface area (BSA) of 306 Å) and outer domain (residues 419, 421–423 and 432–441, BSA of 403 Å) of gp120 with 427-Å surface area buried directly at the assembled bridging sheet (Fig. 1b, Additional file 1: Table S1). The N12-i2 footprint within the M48U1-triggered gp120 also involves residues at the base of the V3 loop (residues 298–301 and 324–327, BSA of 229 Å) that include a GDIR sequence motif known to be engaged in the CCR5 co-receptor binding and recognized as a part of the prominent site of antibody vulnerability of HIV-1 Env targeted by multiple broadly neutralizing antibodies (bnAbs) directed to the N332 high-mannose patch [47].

### N12-i2 binds by utilizing a new sulfo-tyrosine binding site

N12-i2 contacts the Env antigen almost entirely through its heavy chain (BSA of 996 Å, Fig. 1, Additional file 1: Table S1), with the light chain contacts (BSA of 34 Å) limited to two interactions mediated through CDR L1 (Fig. 1a, b). From the heavy chain, the majority of the binding is contributed by CDR H3 (BSA of 630 Å, Additional file 1: Table S1) and the two adjacent sulfoTyrs—TYS^100A^ and TYS^100B^ (total BSA of 343 Å), located at the tip of CDR H3 (Fig. 1c). TYS^100A^ and TYS^100B^ are critical for N12-i2 attachment to Env antigen providing approximately one third of N12-i2’s BSA and form two anchor points that bridge the inner and outer domains of gp120 (Fig. 1a, c, density for the two sulfo-tyrosine binding sites is shown in Additional file 2: Figure S1). Interestingly, contact residues that form the new sulfo-tyrosine binding site, TYS^100B^, are highly conserved, more conserved even than those forming the pocket previously defined by 412d. The consensus sequence, illustrated in Fig. 1d, shows the conservation of sequence between approximately 32,000 sequences from the HIV-1 sequence database compendium, a sequence database of aligned HIV-1 sequences, using the program WebLogo [42].

**Fig. 2:**
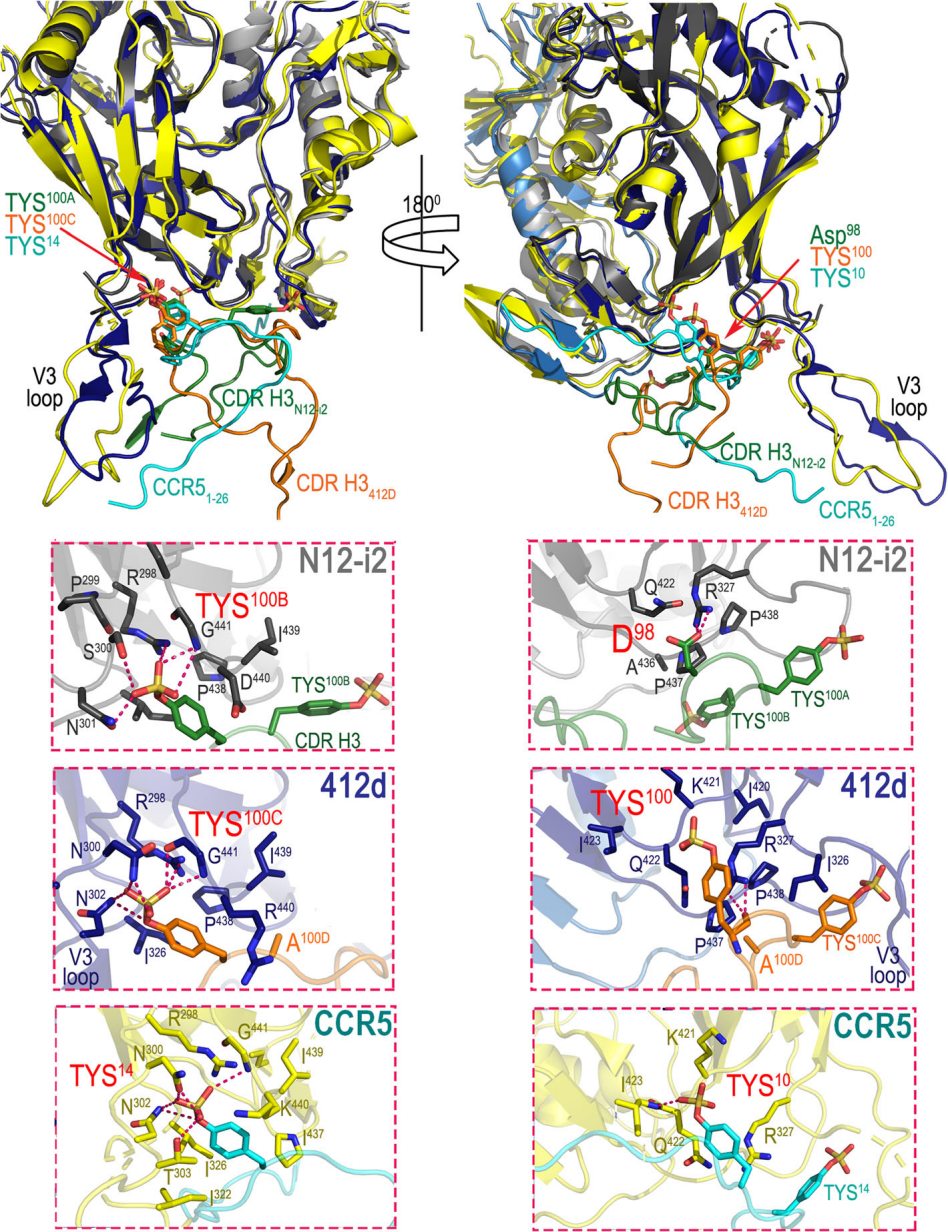
Molecular details of sulfoTyr (TYS) interactions with antibodies N12-i2, 412d, and the CCR5 co-receptor. N12-i2, CCR5, and 412d utilize two TYSs in gp120 binding (N12-i2: TYS^100A^ and TYS^100B^; CCR5: TYS^10^ and TYS^14^ (PDB code: 6MET), and 412d: TYS^100^ and TYS^100C^ (PDB code: 2QAD) with one (TYS^100A^ of N12-i2, TYS^14^ of CCR5, and TYS^100C^ of 412d) occupying the same binding pocket. The right panel shows the binding mode of TYS in the common binding pocket. TYS^100A^, TYS^14^, and TYS^100C^ bind in a similar manner with the sulfate coordinated by hydrogen bonds (H-bonds) to Arg^298^, Ser^300^ (Thr^303^ in CCR5 and Asn^300^ in 412d), Asn^301^ (Asn^302^ in CCR5 and 412d) and to the main chain of Gly^441^. In addition, the aromatic ring of TYS is involved in hydrophobic interactions with Ile^326^ and Ile^439^, and hydrophobic and polar cation-π interactions with Pro^438^ and Asp^440^ (Lys^440^ in CCR5 and Arg^440^ in 412d). The 180° view shows the details of Asp^98^ in N12-i2 which occupies the same binding pocket as the second TYS of CCR5 and 412d (TYS^10^ and TYS^100^). Asp^98^ of N12-i2 buries 19 Å^2^ BSA in the N12-i2 Fab-gp120_93TH057_ core_e_-M48U1 complex and thus contributes significantly to N12-i2 binding. Asp^98^ forms a salt bridge to Arg^327^ with the aliphatic part of its side chain packing against Pro^437^ and Pro^438^. TYS^10^ of CCR5 forms H-bonds with the main chains of Ile^423^ and Gln^422^ and a salt bridge with Lys^421^ and Arg^327^. TYS^100^ of 412d is engaged in a main chain H-bond to Arg^327^ with its aromatic ring packed against the guanidinium group. TYS^100^ also forms a H-bond with Gln^422^ and a salt bridge with Arg^419^. The differences in this second sulfo-tyrosine binding pocket in CCR5 and 412d may be due to gp120 sequence differences in this region. gp120 numbering is relative to the HXBC2 reference sequence

TYS^100A^ occupies the binding site within the gp120 outer domain at the V3 base, which overlaps the binding site for TYS^14^ of the CCR5 co-receptor [21] and TYS^100C^ of 412d [34], the only other TYS containing CoRBS antibody whose structure has been determined and described in complex with the gp120 antigen. This TYS binding site is characterized by sulfate hydrogen bonds with Arg^298^ and the main chain of Gly^441^ as well as several gp120 sequence-dependent H-bonds and hydrophobic Van der Waals contacts to the TYS aromatic phenyl ring with Ile^326^, Ile^439^, and Pro^438^ (HXBC2 gp120 sequence numbering) (Fig. 2).

**Fig. 3:**
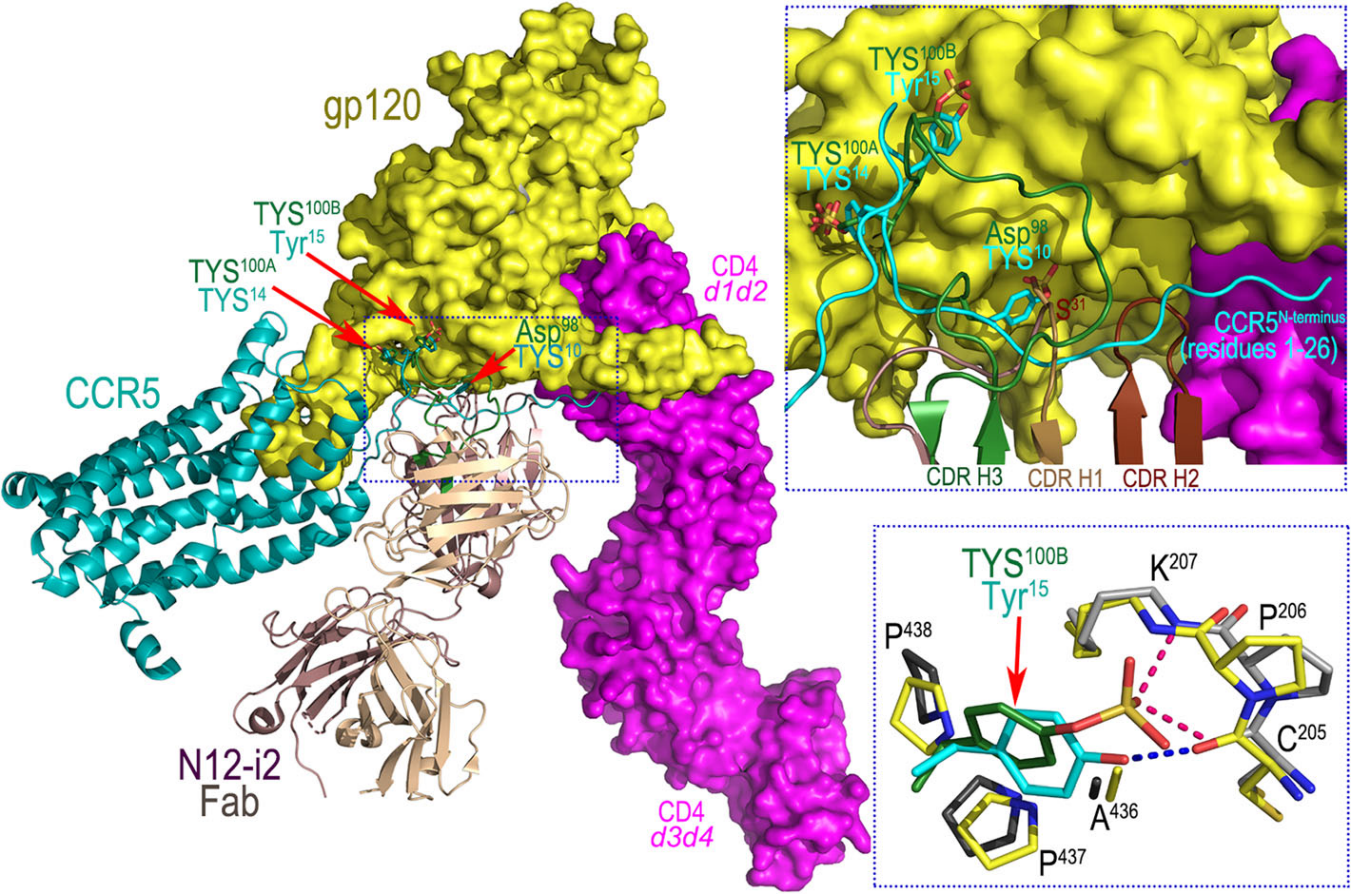
Comparison of binding modes of N12-i2 and CCR5 co-receptor to HIV-1 Env gp120. N12-i2 Fab-gp120_93TH057_ core_e_-M48U1 and CCR5-gp120-CD4d1d4 (PDB code 6MET) complex structures are superimposed based on gp120 outer domain. The N12-i2 Fab is displayed over the CCR5-gp120-CD4d1d4 complex (CCR5 shown as ribbon diagram and the molecular surface is displayed over gp120 and CD4). The TYS of N12-i2 and CCR5 are indicated by red arrows. The blow-up views show details of the TYS binding sites. The hydrogen bond network formed at TYS^100B^ of N12-i2 and Tyr^15^ of CCR5 is shown with dotted lines

Interestingly, TYS^100B^ of N12-i2 utilizes a previously unknown binding pocket formed within the outer and inner domains proximal to the bridging sheet (Fig. 1c). The TYS^100B^ binding site involves residues of the β21-β22 connecting coil (outer domain) and the β3-β4 connecting coil (inner domain) and thus is formed at the base of the bridging sheet on both the inner and outer domain halves (Fig. 1c, blowup). Hydrophobic contacts and two hydrogen bonds to the main chain atoms of Cys^205^ and Lys^207^ contribute to the TYS^100B^ binding within its gp120 pocket. The newly defined pocket for TYS^100B^ of N12-i2 represents a third possible binding site for TYS within the CoRBS which perfectly aligns with the anchor site for Tyr^15^ and potentially TYS^15^, if modified on CCR5. Figure 3 shows the superimposition (based on the gp120 core molecule) of structures of N12-i2 Fab-gp120_93TH057_ core_e_-M48U1 and recent cryo-electron microscopy CCR5-gp120-CD4*d1d4* (PDB code 6MET, [21]) complex. N12-i2 and CCR5 approach gp120 from different angles; N12-i2 occupies the gp120 regions more proximal to the CD4 binding site whereas CCR5 binds at the V3 loop region. Although the N12-i2 and CCR5 attachment sites do not directly overlay, N12-i2 effectively mimics the CCR5 N-terminus interaction within the CoRBS. Of the three CDRs of N12-i2 heavy-chain binding in this region, CDR H3 is the major anchoring point, placing its TYS^100A^ and TYS^100B^ within the binding pockets of Tyr^14^ and Tyr^15^ of CCR5 (Fig. 3, blow-up view). CDR H1 and CDR H2 serve as replacements for much of the remainder of the CCR5 N-terminus. Interestingly, TYS^100B^ binds gp120 almost identically to Tyr^15^ of CCR5 (Fig. 3, blowup) with two strong H-bonds to gp120 versus the one from CCR5. Since Tyr^15^ in CCR5 may be also sulfo-tyrosinated [36], albeit at a lower frequency than Tyr^10^ or Tyr^14^, the binding of N12-i2 TYS^100B^ likely serves as a model for TYS^15^ binding in CCR5.

Although N12-i2 engages two sulfoTyrs for binding, their contribution to the interface is not equal. TYS^100A^ buries 211 Å^2^ of surface area in the complex, and its aromatic ring is tightly sandwiched on both sides by hydrophobic residues with its sulfate making rich network hydrogen bonds to gp120 main and side chain atoms (Fig. 2). In contrast, TYS^100B^ has a BSA of 130 Å^2^ and one face of its aromatic ring is exposed to solvent with the other buried at the complex interface. This BSA is almost identical to Tyr^15^ of CCR5; 136 Å, with the main difference between the two being the added H-bond in N12-i2 TYS^100B^. In addition to TYS^100A^ and TYS^100B^, which provide the two major attachment points for N12-i2 in its binding to the positively charged surface of gp120 (Fig. 1c, blowup), there are other important H-bond interactions formed at the complex interface. These include Arg^26^ of CDR H1, which establishes a network of H-bonds to the main chain atoms of Gly^324^ of the V3 base, and Asp^98^ of CDR H3 which is involved in salt bridge interactions with Arg^327^. Interestingly, Asp^98^ occupies the same binding pocket as the second TYS of 412d, TYS^100^, which is analogous to TYS^10^ in CCR5, and contributes significantly to the complex interface by establishing a salt bridge and packing the aliphatic part of its side chain against Pro^437^ and Pro^438^, at the base of the bridging sheet (Fig. 2).

### The N12-i2 epitope overlaps epitopes recognized by other CoRBS antibodies in binding to Env

**Fig. 4:**
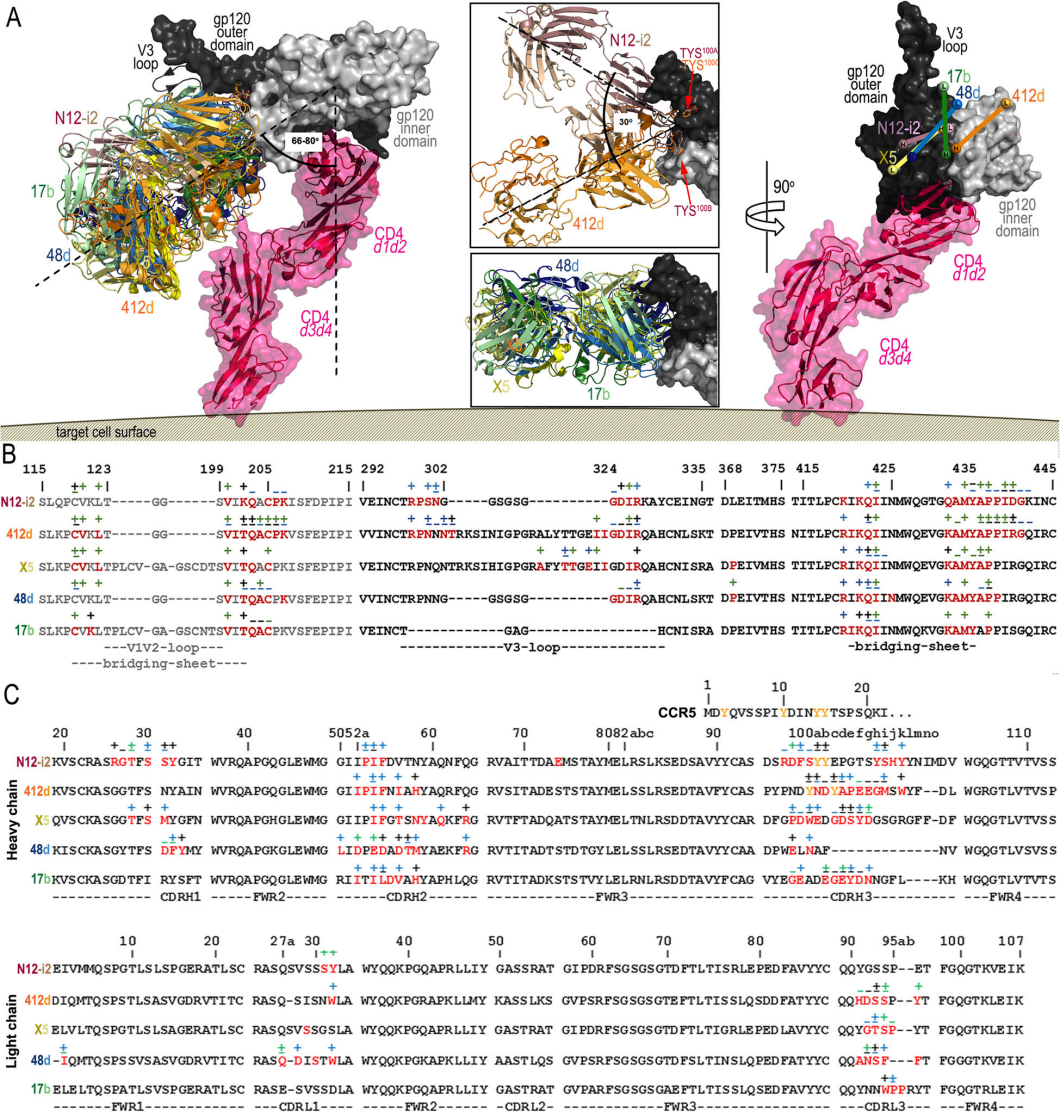
Binding of N12-i2 and other CoRBS antibodies to HIV-1 Env. **a** Overlay of N12-i2 and CoRBS antibodies, 412d (PDB ID 2QAD), X5 (2B4C), 48d (4DVR), and 17b (1GC1), bound to gp120 oriented relative to the target cell membrane. The structural alignment based on the gp120 outer domain of the Fab 412d-gp120_YU2_ core-CD4 complex (PDB ID 2QAD) and the target cell receptor CD4 extracellular portion modeled by superimposition of the d1-d2 domains to the unliganded CD4 (d1-d4 CD4, PDB code: 1WIO). An alternate conformation of the V3 loop seen in the Fab X5- gp120_JR-FL_ core-CD4 complex (PDB code: 2B4C) is shown as a ribbon. The blow-up views show the alignment from the top (180° vertical rotation showing orientations of N12-i2 and 412d (top) and 48d, X5, and 17b (bottom). Angles were calculated using the center of mass of gp120 and the CD4 d1 domain to define a vector perpendicular to the target cell membrane and the mAb CDR residues to define vectors for the mAbs. A 90° view displays the relative positions of each mAb to CD4-triggered gp120. Heavy- and light-chain (V_h_ and V_L_) positions determined by the center of mass of their CDRs (displayed as balls). **b** Epitope footprints. gp120 buried surface and contact residues shown over the primary gp120 sequence of the isolates used in co-crystallization studies. **c** Fab interface residues with the N-terminal sequence of the CCR5 co-receptor aligned above the CDR H3 sequence. Tyrosines modified by post-translational *O*-sulfation are shown in yellow. In **b** and **c**, residues buried at the surface as determined by PISA (http://www.ebi.ac.uk/msd-srv/prot_int/cgi-bin/piserver) are shown in red and contact residues as defined by a 5-Å distance cutoff are shown with interactions, main chain (−), side chain (+), or both (±), colored based on contact type: hydrophobic (green), hydrophilic (blue), or both (black)

Several other CoRBS antibodies have been isolated and characterized to date, mainly in the context of direct neutralizing activities [9, 29, 30, 32–35]. However, the molecular basis of their interaction with the Env antigen at the atomic level has only been described for four: 412d, 17b, 48d, and X5, crystallized in complex with either the CD4-triggered gp120 core (17b, 48d) or the CD4-triggered gp120 core with the V3 loop added (412d, X5) [12, 32–34]. Interestingly, of these 5 CoRBS antibodies, only 48d does not use the VH1-69 gene for the heavy chain, but instead uses VH1-24. Among them, only 412d is similar to N12-i2 in its involvement of two sulfoTyrs (TYS^100^ and TYS^100C^) and a 21-residue long CDR H3 for engagement of CD4-triggered gp120. X5 uses a 22-residue long CDR H3 for binding and has also been reported to be tyrosine sulfated, but unlike 412d, this sulfation is not required for the binding to gp120 [33]. 17b and 48d are not modified by *O*-sulfation and utilize relatively short CDR H3s (17b and 48d of 19- and 10-residue long, respectively). We compared modes of binding as well as epitope footprints of N12-i2 to the other CoRBS antibodies for which co-crystal structures are available (Fig. 4). Analysis of epitope footprints (Fig. 4b) indicated that N12-i2 shows the closest similarity to 412d, with an epitope footprint almost exactly mirrored by N12-i2 with only a few more contacts at the base of the V3 loop. X5 also bears some similarities to N12-i2 but contacts more elements of the V3 loop and has significantly less contact within/proximal to the assembled bridging sheet. Although N12-i2 was co-crystalized with the gp120 core_e_, where no V3 loops are present, the analysis of its mode of binding excludes the possibility of additional contacts within the V3 loop region (Figs. 1 and 3a). The antibody footprints of 48d and 17b are the most dissimilar to N12-i2 with several contacts missing at the V3 loop base and slightly fewer interactions with the bridging sheet (Fig. 4b). The extent of each antibody footprint is reflected well by the buried surface area (BSA) of each complex. The interface of 412d is the most extensive with a BSA of 2377 Å^2^ followed by N12-i2 with BSA of 2038 Å^2^, 48d of 1993 Å^2^, X5 of 1729 Å^2^, and 17b of 1132 Å^2^. The relatively small interface of the X5 complex is in spite of the extensive contacts of X5 to the V3 loop.

Although the gp120 contact residues are largely shared among N12-i2 and other CoRBS antibodies, their bound orientations show noticeable differences, particularly when analyzed in an axis parallel to the target cell membrane (Fig. 4). As shown in Fig. 4a (blow-up views), the modes of binding in the parallel axis differ significantly with N12-i2 and 412d being the most variable (a 30° angle difference as calculated for the FWRH2). N12-i2 and 412d approach CD4-triggered gp120 from different angles even though they both utilize the same sulfo-tyrosine binding site on gp120 (TYS^100A^ of N12-i2 and TYS^100C^ of 412d) for one of the two TYSs of their CDR H3. The attachment points for the second TYS (TYS^100B^ of N12-i2 and TYS^100^ of 412d) are located on two opposite sides of gp120, at the inner-outer domain interface in the N12-i2 complex and at the V3 base in the 412d complex, most likely determining the angle by which these two antibodies engage with the Env antigen. Less pronounced differences are noticed in an angle of approach in an axis perpendicular to the target cell membrane (the axis of the d1-d2 domain of CD4 attached to gp120). N12-i2 and all other CoRBS antibodies bind at a similar angle in the perpendicular axis with X5 positioned the closest (64^o^ angle) and 412d binding at the largest angle (80°) as measured by the average CDR position relative to the CD4-d1 domain with the center of gravity of gp120 used as the origin.

All antibodies, with the exception of X5, place the heavy chain proximal to the CD4 binding site (Fig. 4a, right panel). Also, the binding of these five antibodies is mostly determined by their heavy chains with N12-i2 being the most heavy-chain dependent with only two contacts contributed by its light chain. mAb 48d has the largest contribution to binding from its light chain; 35%, followed by 17b with 15% (Fig. 4a, right panel).

### ADCC activities of N12-i2 and other CoRBS antibodies correlate with antibody binding levels

**Fig. 5:**
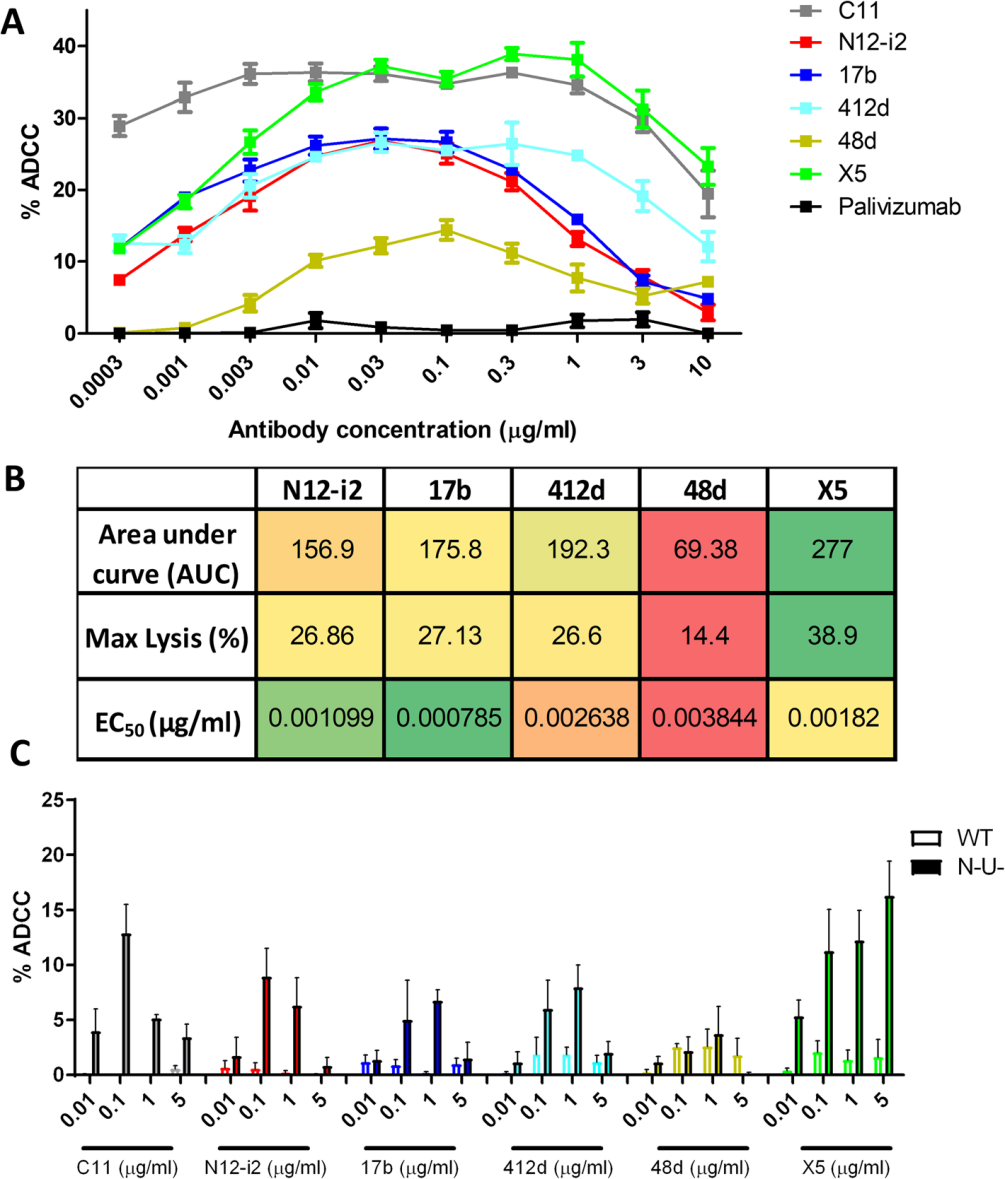
ADCC to gp120-coated and ADA-infected target cells. **a** RFADCC using gp120-coated GFP-CEM-NKR-CCR5-SNAP target cells over a range of antibody concentrations (0.0003–10 μg/ml) displayed as % killing. *n* = 3, mean ± SEM. Anti-Cluster A antibody C11 and palivizumab were used as positive and negative controls, respectively. **b** Heat map showing the ADCC activity measures as area under the curve (AUC), maximum lysis and EC50 for all five CoRBS antibodies in the RFADCC assay. **c** ADCC against pNL4/ADA and pNL43/ADA/N-U- infected CEM-NKR target cells over a range of antibody concentrations. *N* = 3, mean + SEM, individual data is displayed in Additional files 7 and 8

Structural analyses indicated that antibodies targeting the CoRBS may or may not utilize TYSs for binding and showed all antibodies bound by different modes of attachment, as indicated by the exact heavy light chain contact surfaces (Fig. 4). We aimed to determine if the differences in fine epitope specificities and modes of attachment were reflected in how the target epitopes were recognized at the antigen-coated or infected cell surface and if the antibody binding levels correlated to Fc-effector function. We selected two different ADCC measurements: RFADCC activity in a model of gp120 BaL coated GFP-CEM-NKR-CCR5-SNAP target cells [48] and a model of CEM-NKR cells infected with a wild type or a Nef- and Vpu-defective virus [10]. The RFADCC system was selected because of its simplicity and to avoid potential complications of dynamics of target populations in the virion-bound or infected cell systems [49, 50]. In contrast, the ADA infected model is more reflective of in vivo conditions and allowed us to model the complexity of Env targets on the infected cell surface. ADCC percent lysis curves for all CoRBS antibodies in the RFADCC assay are shown in Fig. 5a, along with detailed results for important parameters of ADCC, area under the curve (AUC), maximum lysis (%), and the mAb concentration which achieves 50% of the maximal lysis (EC50) of the tested antibodies, displayed in a heat map in Fig. 5b. All CoRBS antibodies mediated significant cytotoxicity above the palivizumab-negative control with maximum lysis levels for 48d peaking at 14.4 ± 1.37%, 412d at 26.6 ± 1.9%, N12-i2 at 26.86 ± 0.37%, 17b at 27.13 ± 1.99% and the most potent inducer of RFADCC, X5 peaking at 38.9 ± 0.85%. N12-i2 showed the second best EC50 among the CoRBS antibodies with only 17b requiring a lower concentration of antibody to achieve half maximal binding. Interestingly, a similar pattern of ADCC levels between the CoRBS antibodies was observed in a model of CEM-NKR cells infected with a Nef- and Vpu-defective virus, known to be susceptible to ADCC responses mediated by CD4i Abs [10, 51, 52] (Fig. 5c). As expected, we observed a significant correlation between the two ADCC assays used (Additional file 3: Figure S2 A, B) [53], due to the fact that in both assays gp120 is stabilized in the CD4-bound conformation. In the RFADCC assay, gp120 attaches to cell surface CD4 and stabilizes its CD4-bound conformation. Similarly, in the infected cell assay, cells are infected with viruses lacking Nef and Vpu, which downregulate CD4, leading to an accumulation of CD4 at the cell surface that interacts with Env to generate the CD4-bound conformation [10]. Altogether, these results indicate that the tested antibodies behaved similarly, with X5 resulting in the most cell cytotoxicity, 48d the lowest, and N12-i2 and the other CoRBS antibodies moderate ADCC. As previously reported, target cells infected with wild type virus coding for Nef and Vpu were resistant to ADCC.

**Fig. 6:**
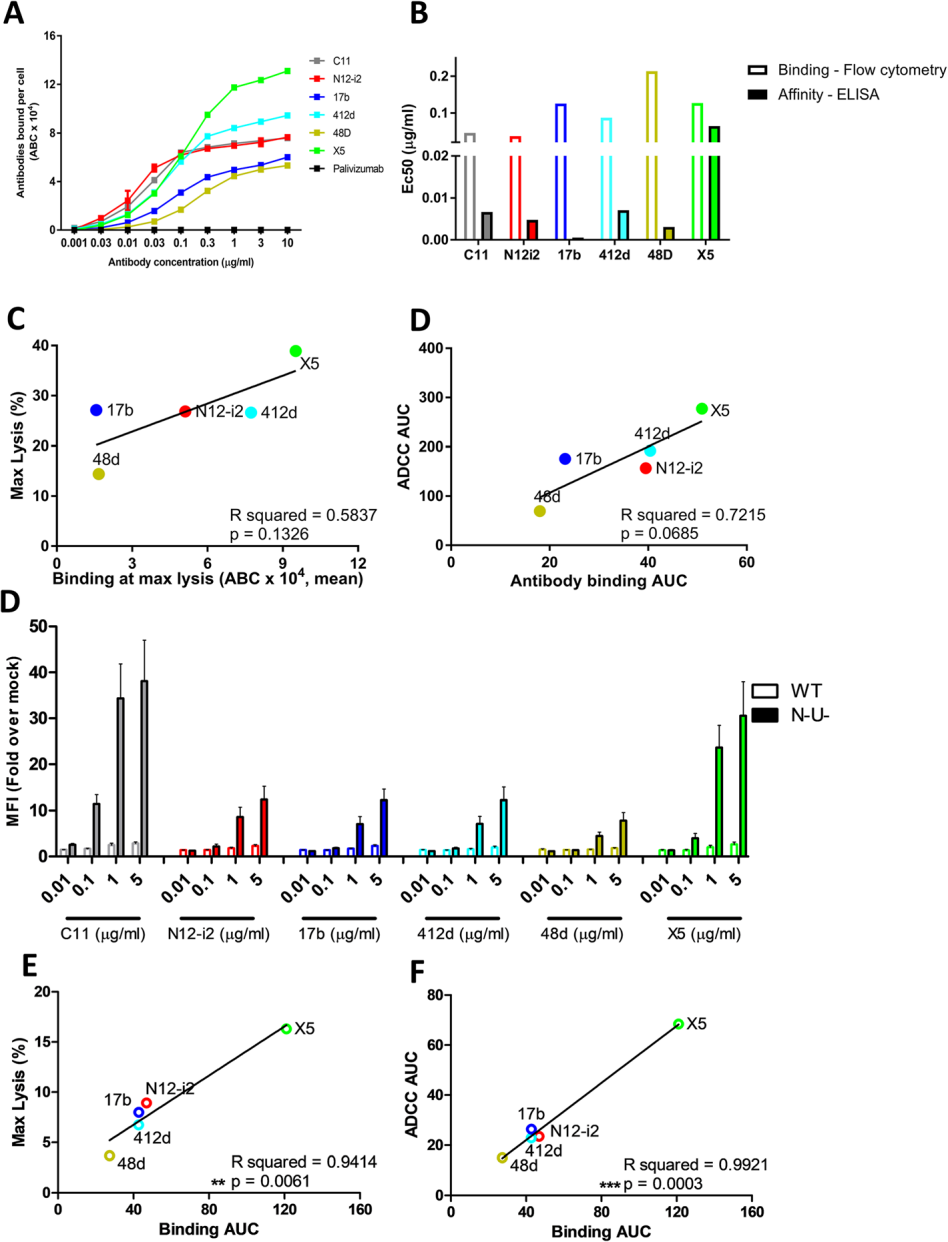
Correlations between antibody affinities, binding levels, and ADCC activity. **a** Number of antibodies bound per cell to GFP-CEM-NKR-CCR5-SNAP target cells coated with gp120 BaL. Quantum MESF beads were used to quantify the number of AF647-labeled antibodies bound per cell (ABC) to GFP-CEM-NKR-CCR5 target cells coated with gp120 BaL. *n* = 3, data is mean ± SEM. **b** EC50 of antibodies bound per cell to GFP-CEM-NKR-CCR5-SNAP target cells coated with gp120 BaL (hollow bars) and EC50 of antibody binding to full-length single chain (FLSC) (solid bars). **c** Correlations between the antibody binding levels to GFP-CEM-NKR-CCR5-SNAP target cells coated with gp120 BaL and ADCC parameters: maximum lysis vs. the number of antibodies bound at the concentration resulting in maximum lysis (left panel), area under the curve (AUC) of ADCC vs. antibodies bound (right panel) (**d**) Antibody binding to pNL4/ADA and pNL43/ADA/N-U- infected CEM-NKR target cells over a range of antibody concentrations. *n* = 3, data is mean ± SEM. **e** Correlations between the antibody binding levels to NL43/ADA/N-U- infected cells and ADCC parameters: ADCC maximum lysis vs. binding area under the curve (left panel), NL43/ADA/N-U- ADCC area under the curve vs. binding area under the curve (right panel). **P < 0.01, ***P < 0.001 via a two-tailed Pearson correlation. Individual data is supplied in Additional files 9 and 10

Since variable ADCC activities were observed for CoRBS antibodies recognizing largely overlapping epitopes, we wanted to determine if a difference in the overall antibody affinity or number of antibodies bound per cell could account for this. To compare binding of CoRBS antibodies to gp120-coated cells, antibodies were labeled with AF647, analyzed by flow cytometry, and the total number of antibodies bound per cell (ABC) calculated over a range of concentrations. Following incubation with gp120-coated GFP-CEM-NKR-CCR5-SNAP target cells, all CoRBS antibodies showed significant binding above the palivizumab-negative control with 48d having the fewest ABC and X5 the most (Fig. 6a). Antibody affinity for full-length single chain antigen (FLSC-protein consisting a covalent dimer of gp120 and CD4, [27]) was assessed as a model of affinity towards the CoRBS and EC50 values are displayed alongside EC50 values of ABC in Fig. 6b. Affinity EC50 was very similar between CoRBS antibodies, with the exception of X5 which had the highest EC50 for both affinity and ABC, indicating the poorest affinity for the region but highest level of binding at high concentrations. There was no positive correlation between the AUC of antibodies bound per cell and antibody affinity for FLSC (Additional file 3: Figure S2 C, D). A trend towards significance was seen between the maximum lysis of target cells and the number of antibodies bound per cell at the antibody concentration leading to maximum lysis as well as between overall antibody binding and ADCC, defined by area under the curve (Fig. 6c, d), suggesting that the level of antibody binding could be an important determining factor for the level of cell cytotoxicity [53, 54]. A similar pattern of antibody binding was observed between gp120-coated and ADA/N-U- infected cells with X5 binding the most, 48d the least, and N12-i2, 412d, and 17b with moderate binding (Fig. 6e). Interestingly, while the level of binding did not significantly correlate to ADCC in the gp120-coated cell model, both area under the curve and maximum lysis ADCC parameters were significantly associated with the level of antibody binding in ADA/N-U- infected cells (Fig. 6f, g).

### ADCC activities of N12-i2 and other CoRBS antibodies correlate with the angle of the Fc in relation to the target cell surface

**Fig. 7:**
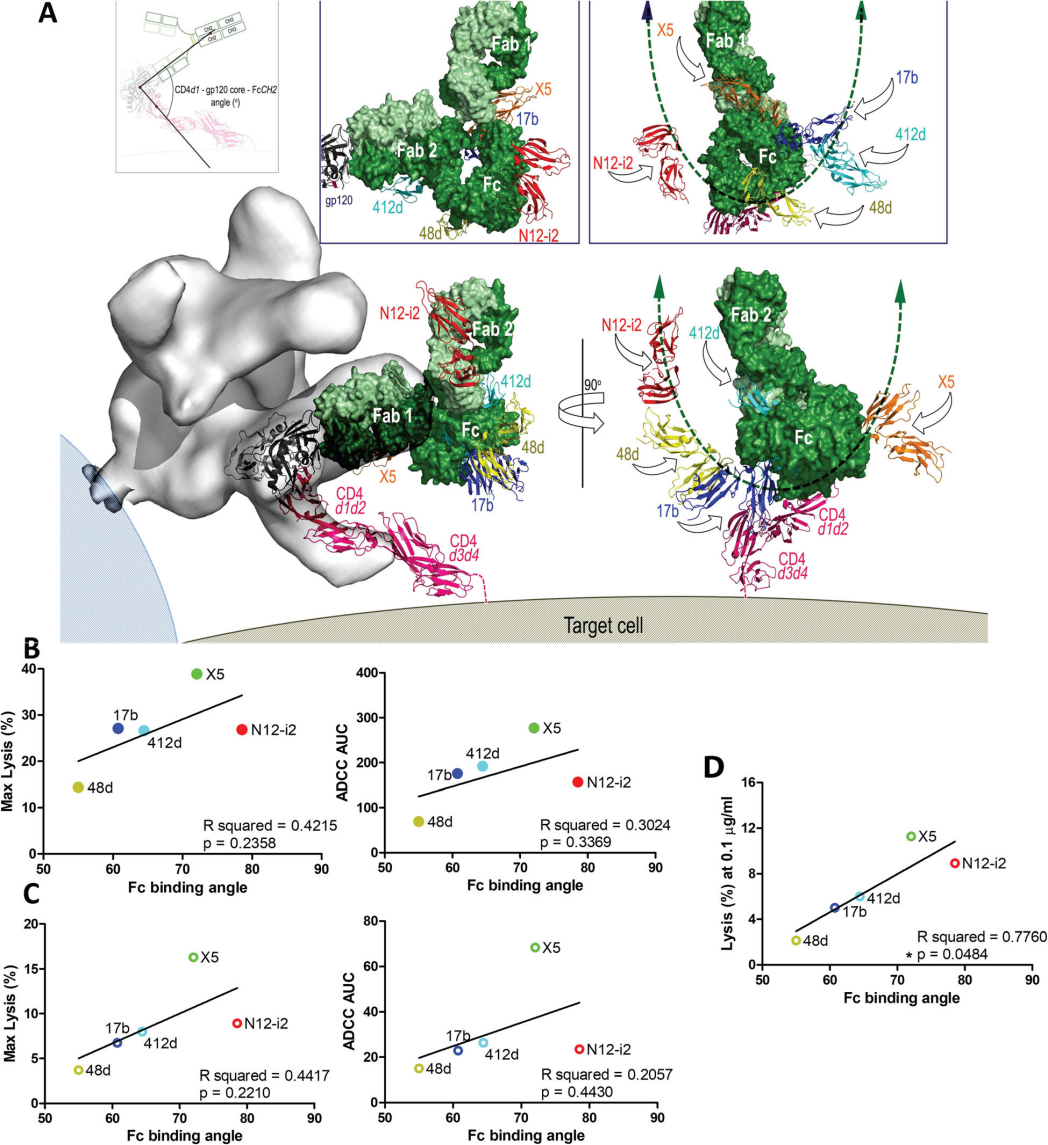
Correlations between angle of the Fc in relation to target cell surface and ADCC activities. **a** Models of putative immune-complexes formed by CoRBS antibodies at the target/effector cell interface were assembled as described previously [55]. Virion position modeled based on a cryo-EM structure of 17b bound to a d1-d2 CD4-triggered Env trimer. Four-domain CD4 was generated by superimposing four-domain CD4 (PDB code: 1WIO) onto d1-d2 domains of CD4 in the complex, 17b IgG was built by superimposition of monoclonal murine antibody subclass IgG1 (PDB code 1IGY) onto the 17b Fab (space fill model, green). There are two possible orientations: the main figure shows the complex formed by engagement of Fab 1 with the alternate complex formed through Fab 2 in the box above each view. Human FcγRIII receptor (residues 1–172) was added by overlaying the Fc-FcγRIII complex (PDB code 1E4K) onto Fc domain of the modeled IgG (ribbon diagram, blue for 17b). Analogous models were built for N12-i2, 412d, X5, and 48d for putative position of the human Fcγ receptor bound to Fc in the immune-complexes, for clarity only the FcγRIII receptors are shown; red: N12-i2, cyan: 412d, orange: X5 and yellow: 48d. The left panel shows the putative position of the effector cell with angle of attack by which the effector cell targets the Fc region of each mAb, shown by arrows. Range of FcγIII receptor positions depending on modeled mAb is indicated by a green double-sided arrow. Both orientations of the IgG place the FcγRIII receptor bound to N12-i2 (red) in a favorable orientation for Fc-effector cell access. **b** Correlations between Fc binding angle ADCC—maximum lysis (left) and AUC (right). **c** Correlation between Fc binding angles and ADCC of NL43/ADA/N-U- infected cells—maximum lysis (left panel) and AUC (right panel). **d** Correlations between Fc binding angles and % lysis of NL43/ADA/N-U- target cells at concentration of 0.1 μg/ml. *P < 0.05 via two-tailed Pearson correlation

It has previously been suggested that altering the binding orientation of an antibody, and therefore changing the angle of the Fc in relation to the target cell surface, can alter the ADCC potency of a given antibody [55, 56]. To examine if the antibody binding mode and Fc exposure contributed to the effectiveness of Fc-mediated responses directed at the CoRBS region, we estimated the angle between the target cell surface and the Fc of N12-i2 and the other CoRBS antibodies (Fig. 7a). In each case, a model was prepared of the whole IgG1 bound to gp120 (using the crystallographic structure of mouse IgG1, PDB ID 1IGY, and the respective Fab-gp120 complex structure). 1IGY was selected as it has the most typical IgG conformation of the available structures with Fc-Fab hinge angles of 78° and 123° as compared to the mean value of 107° ± 30°. Since the IgG molecule is asymmetric with different Fab-Fc angles for each IgG arm, we modeled complexes formed using Fab1 and Fab2 of immunoglobulin and averaged the positions of the C_H_2 domains from each of the two orientations. Angles were calculated using the center of mass of gp120 and the center of mass of the CD4 d1 domain to define a vector perpendicular to the target cell membrane. N12-i2 had the largest average Fc angle from the cell surface (78.6°), followed by X5 (72.1°), 412d (64.5°), 17b (60.8°), and finally 48d with the smallest binding angle relative to the cell surface, 55° (Fig. 7a). A trend towards correlation was observed between the Fc angles and both max lysis and ADCC AUC in both the gp120-coated RFADCC and ADA N-/U- infected cell assays (Fig. 7b, c). However, although a positive trend towards correlation was clear for each parameter tested, none reached statistical significance. Interestingly, analyses of the correlation between lysis and the Fc angle at each concentration tested (Additional file 5: Figure S4) indicated that there was a statistically significant correlation (*p* = 0.0484) at an antibody concentration of 0.1 μg/ml (Fig. 7d) in the ADA N-/U- infected cell assay. This was not observed at any other antibody concentration in both ADCC measurements (Additional file 5: Figure S4, Additional file 6: S5).

## Discussion

Antibody-dependent cellular cytotoxicity is a mechanism whereby antigen-antibody complexes present on target cells arm effector cells (typically NK cells or macrophages), enabling them to lyse target cells. Multiple variables contribute to the effectiveness of ADCC mediated by a given antibody including epitope specificity, affinity for the antigen, and geometry of the formed antigen-antibody-effector cell complex [55–57]. Several lines of evidence indicate that antibodies recognizing epitopes which emerge on the viral envelope trimer when it transitions from native, unliganded, to the open CD4- triggered state in the entry process or within Env conformations available on HIV-1-infected cells are capable of some of the most efficient Fc-effector cell-mediated functions against HIV [10, 15, 50]. One example of these potent ADCC targets are the CD4i epitopes within the C1C2 region of gp120, known as Cluster A epitopes [56, 58, 59]. These epitopes are strictly CD4 dependent and require structural rearrangements of the Env trimer post-CD4 binding for exposure and, when exposed, constitute favorable targets for antibodies capable of potent ADCC activities. Interestingly, our previously published data indicate that not all antibodies recognizing overlapping epitopes in the Cluster A region are capable of the same potent ADCC and that the mode of antibody attachment may contribute to the efficiency of Fc-effector responses to these epitope targets [52, 55].

Here, we describe the molecular details of the Env antigen interaction and Fc-mediated effector function of mAb N12-i2, an antibody specific for an epitope within another Env CD4i epitope region, the co-receptor binding site. N12-i2 was isolated from an individual in a cohort of Natural Virus Suppressors (NVS) and was found to be an effective neutralizer of tier 1 viruses and potent inducer of ADCC in an assay format using gp120- or virion-sensitized target cells [15]. To date, several antibodies specific for CoRBS epitopes have been described and characterized at the molecular level, but mostly in the context of their direct neutralizing activities [9, 15, 29, 30]. To fill this gap in understanding ADCC responses to CoRBS epitopes, we evaluated the ability of N12-i2 and other CoRBS antibodies with known structures to lyse cells infected with the ADA virus or coated with BaL gp120 to determine if the fine epitope specificity and mode of antibody attachment to epitopes within the CoRBS contribute to the effectiveness of ADCC response as they do to Cluster A Env targets.

The previously determined unbound structure of the N12-i2 Fab revealed only those properties of the antigen-binding site that are shared with other CD4i mAbs recognizing CoRBS-associated epitopes, i.e., a long (25-residue) CDR H3, an acidic paratope (− 2 net charge as calculated based on the unmodified amino acids), and a tyrosine-sulfated CDR H3 [15]. The unbound structure of the Fab fragments provided no information on which tyrosines were sulfated or how they might interact with gp120; also, the tyrosine-containing region of the CDR H3 tip was disordered and not resolved in these structures. The N12-i2 Fab-gp120_93TH057_ core_e_-M48U1 complex structure presented here reveals that two tyrosine residues occupying adjacent positions at the tip of CDR H3 are sulfated (TYS^100A^ and TYS^100B^) and both contribute to the gp120 binding by providing a significant portion of the buried surface area of the interface. Interestingly, whereas the TYS^100A^ of N12-i2 occupies the pocket defined previously as the binding site for TYS^100C^ of 412d [34] and TYS^14^ of co-receptor CCR5 [21] (Fig. 1 and Additional file 2: S1), the TYS^100B^ of N12-i2 binds by docking to a new pocket. This new TYS binding pocket is formed by residues of high sequence conservation among HIV-1 clades. It is known that tyrosines at positions 3 and 10 and adjacent positions 14 and 15 of CCR5 can be modified by post-translational *O*-sulfation with a minimum of two (10 and 14) required to mediate HIV-1 cell entry [36]. The recent cryo-electron microscopy structure of CCR5-gp120-CD4*d1d4* (PDB code 6MET [21]) complex as well as modeling tests with the NMR structures of peptides corresponding to CCR5 N-terminus [34, 60] provided information about binding sites on gp120 of TYSs at positions 10 and 14 of CCR5 but not 3 and 15 (Fig. 3). The N12-i2 Fab-gp120_93TH057_ core_e_-M48U1 complex structure indicates that two adjacent TYS binding sites capable of accommodating TYS at positions 14 and 15 of CCR5 could be formed at the surface of CD4-triggered gp120 and therefore for the first time defined the possible docking site for TYS^15^ of CCR5. Interestingly, reconstitution of HIV-1 entry with an N-terminal deletion mutant, Δ2-17, by sulfated CCR5 peptides as well as mutagenesis studies implicate positions 10 and 14 as being the most important sites for sulfation [34] but sulfation at positions 10, 14, and 15 are required to approach wild type entry rates [61]. This is in agreement with the inferred sulfation pattern of CCR5 by human tyrosylprotein sulfotransferases (TSPT) 1 and 2 which suggests that tyrosines 14 and 15 are sulfated before 10 in the absence of a methionine at position 1 [62] and that tyrosine 3, then 14 and 15, and finally 10 are sulfated in the presence of a methionine at position 1 [63].

To date, several CoRBS-specific antibodies have been isolated [9, 29, 30, 32–35] and four (17b, 412d, 48d, X5) with structures available in complex with CD4-triggered gp120 antigen [12, 32–34]. Not surprisingly, structural analysis indicates that the N12-i2 epitope footprint overlaps with the binding footprints of all four of these antibodies sharing the greatest overlap to the epitope footprint of 412d, the only other CoRBS antibody containing two TYS residues in its CDR H3 that also significantly contribute to its binding (Additional file 1: Table S1). Interestingly, structural comparison highlighted noticeable differences in the bound orientation of N12-i2 Fab and the Fabs of the other CoRBS antibodies as measured by binding angles in an axis parallel to and perpendicular to the target cell membrane (the axis of the d1 domain of CD4 attached to gp120) (Fig. 4). The most pronounced differences were observed in the perpendicular axis with the largest difference in binding between N12-i2 and 412d, a 30° angle difference as calculated from their FWRH2s. This indicates that despite a large overlap in epitope footprint and a similar mechanism of binding involving TYS residues, these antibodies utilize significantly different angles to approach the antigen. With the newly available gp120/CCR5 structure, it becomes clear that N12-i2 more closely mimics the angle of approach of the co-receptor CCR5 (Fig. 3).

The observed differences in bound orientations led us to investigate the potential role of antibody attachment and geometry of the antigen-antibody–effector cell complex in Fc-mediated effector mechanisms to CoRBS epitopes. First, we tested N12-i2 and other CoRBS-specific antibodies for their ability to recognize and mediate ADCC of CEM-NKR cells sensitized with gp120 BaL or infected with ADA virus. Second, to describe how the mode of Fab attachment might contribute to the geometry of the antigen-antibody–effector cell complex, we generated models using the available crystal structures for the inferred CD4-triggered-gp120-antibody-Fcγ receptor complexes formed by N12-i2 and the other CoRBS at the target/effector cell interface and analyzed if the putative angle between the target cell surface and the Fc contribute to the effectiveness of ADCC (Fig. 7). Interestingly, although target presentation may be different on CEM-NKR cells sensitized with HIV-1 BaL gp120 compared to cells infected with the ADA/Nef-Vpu- virus, we observed significant similarity in the measured cytotoxicity of CoRBS antibodies in both models (Additional file 3: Figure S2 A, B). In both assays, X5 provided the most cell cytotoxicity, 48d the lowest level, and N12-i2 and the other CoRBS antibodies moderate ADCC. In agreement with previously published data [10, 37, 38, 64], no cytotoxicity was induced against wild type ADA virally infected cells; CoRBS epitopes, as with other CD4i epitopes, strictly depend on CD4 levels on the infected cell surface and are not exposed on cells with downregulated CD4 [10]. Interestingly, N12-i2 and to some extent the other CoRBS antibodies were able to bind to free BaL and JRFL virions in the absence of a CD4 mimetic (Additional file 4: Figure S3) although Virion recognition was significantly increased in the presence of the CD4 mimetic peptide M48U1. When tested by FCS, N12-i2 showed the highest level of binding to both BaL and JRFL virions (in the absence and presence of M48U1) followed by X5 in binding to BaL and 17b in binding to JRFL (Additional file 4: Figure S3). Noticeably, the observed binding pattern to virions greatly differed from binding to BaL gp120-coated and ADA-infected cells, pointing towards differences in plasticity and conformational flexibility of the Env trimers present on the virions compared to those expressed at the surface of infected cells.

Finally, our data provide evidence that both the level and mode of antibody attachment (which defines the positioning of the Fc domain in relation to the target cell membrane) likely contribute to the effectiveness of ADCC. We observed associations between antibody binding levels and ADCC efficiency in both the Bal gp120-coated and ADA N-/U- infected cell ADCC assays with a statistically significant correlation in the ADA N-/U- infected cell assay but not in the gp120-coated cell format. Furthermore, when the association between effectiveness of lysis and the modeled Fc angle was analyzed separately for each concentration, a statistically significant correlation was observed in the ADA N-/U- infected cell assay only and at a single point of 0.1 μg/ml, a concentration within the peak of the ADCC lysis bell-shaped curve. This indicates that at functional concentrations the mode of antibody attachment may contribute to the effectiveness of Fc-mediated effector mechanism. Antibodies that position their C_H_2 domain in a less favorable orientation (close to the target cell surface) for Fcγ receptor interaction were found to mediate weaker ADCC as compared to those positioning the Fc domain away from target cell surface. However, Fc angle is not fixed due to the flexibility of the hinge region, nor is it the single determinant to the efficiency of ADCC in this study as it can be augmented/diminished by antibody binding level, while antibody affinity did not correlate to ADCC responses. In our CoRBS antibody panel, 48d had the worst ADCC values of all the tested antibodies which coincided with the poorest access to the Fc region in our models as well as the lowest levels of antibody binding. In contrast, N12-i2 had the most favorable access to the Fc region and yet had modest binding and ADCC activity. 48d is also the only antibody tested that does not use the VH1-69 heavy-chain gene, which is associated with Fc-mediated effector functions against gp120 [65].

## Conclusions

Collectively, our data for the first time describes the molecular details of recognition of the epitopes within the co-receptor binding site of the HIV-1 virus. We identified a new TYS binding site which is utilized by N12-i2, an antibody isolated from a subject able to naturally control HIV-1 infection, to efficiently bind to Env. The same binding site may be utilized by HIV-1 to efficiently bind to the co-receptor CCR5. In addition, our data describe potential links between the mode of antibody attachment to the CoRBS and the efficiency of ADCC activity and therefore provides further evidence that the geometry of the antigen-antibody-effector cell complex may contribute to the effectiveness of Fc-mediated effector mechanisms. This suggests that efficiency of antibody binding to a given antigen epitope cannot be the sole determinant for the development of a vaccine based on eliciting an Fc-mediated effector functions. However, how this knowledge can be translated to the development of an Fc-mediated antibody response in the vaccine setting remains to be determined.

## Materials and methods

### Protein purification

The N12-i2 monoclonal antibody (mAb) was purified by HiTrap protein A column (GE Healthcare) chromatography from 293T supernatants prepared by transfecting heavy- and light-chain genes as previously described [15]. The N12-i2 Fab was generated from N12-i2 IgG by papain cleavage according to manufacturer’s protocol (Thermo Fisher) and purified by passage through a protein A column followed by gel filtration chromatography on a Superdex200 16/60 column (GE Healthcare, equilibrated in buffer containing 25 mM Tris-HCl pH 8.5 and 350 mM NaCl). The Fab elution peak corresponding to a molecular weight of approximately 50 kDa was collected and concentrated for use in crystallization trials.

A clade A/E core_e_ gp120_93TH057_ lacking the variable loops prepared as previously described [55] and the CD4-mimetic miniprotein M48U1 were used to prepare the complex with N12-i2 Fab for crystallographic studies [66]. Deglycosylated gp120_93TH057_ core_e_ and the CD4 mimetic peptide M48U1 were mixed at a molar ratio of 1:1.5 to form the complex with N12-i2 Fab and purified by size exclusion chromatography. After concentration, the gp120_93TH057_ core_e_-M48U1 complex was mixed with a 50% molar excess of N12-i2 Fab and passed again through a Superdex200 16/60 column equilibrated with buffer containing 0.15 M NaCl, 25 mM Tris pH 7.0. A small fraction of N12-i2 Fab roughly proportional to the unsulfotyrosinated fraction of N12-i2 measured by mass spectrometry in [15] did not form a complex with gp120. The peak corresponding to the Fab-gp120 complex was concentrated to ~ 10 mg/ml for crystallization experiments. Complex formation and purity were assessed by SDS-PAGE.

### Protein crystallization

Initial screening for crystals of the complex was performed in robotic vapor diffusion sitting trials with Sparse Matrix Screens available from Hampton Crystal Screen (Hampton Research), Precipitant Wizard Screen (Emerald BioSystems), Synergy Screen (Emerald BioSystems) and ProComplex and MacroSol screen from Molecular Dimensions. Crystallization trials were incubated at 22 °C. Screens were monitored periodically to test for protein crystals and, when found, reproduced and optimized using the hanging-drop, vapor diffusion method with drops consisting of 0.5 μl protein and 0.5 μl precipitant solution equilibrated against a 700-μl reservoir at 22 °C.

Crystals of Fab N12-i2-gp120_93TH057_-M48U1 complex were seen in three different conditions: 15% w/v PEG 6000, 5% v/v MPD, 0.1 M MES pH 6.5; 15% w/v PEG 6000, 0.1 M sodium HEPES pH 7.5, 0.1 M potassium chloride; or 20% w/v PEG 8000, 0.1 M sodium HEPES pH 7.0. Cryoprotection for the Fab N12-i2-gp120_93TH057_-M48U1 complex crystals was added with a brief soak of the crystals in mother liquor supplemented with either 15–20% MPD or 25% glycerol. Diffraction quality data was obtained from crystals grown in 15% w/v PEG 6000, 5% v/v MPD, 0.1 M MES pH 6.5, and 65 mM sodium chloride and frozen in liquid nitrogen with 15% MPD as a cryoprotectant.

### Data collection and structure solution and refinement

Diffraction data for Fab N12-i2-gp120_93TH057_-M48U1 was collected at the Stanford Synchrotron Radiation Light Source (SSRL) at the beam line BL12-2 with a PILATUS area detector or on the SSRL beam line BL14-1 with a Rayonix MX325 area detector. The crystals belong to a space group P2_1_2_1_2_1_ with the unit-cell parameters *a* = 52.7, *b* = 69.5, *c* = 213.5 Å and *α* = *β* = *γ* = 90° with 4 molecules and one complex present in the asymmetric unit (ASU). All data were processed and reduced with HKL2000. Two data sets were generated from one crystal from data measured at both SSRL beam lines 12-2 and 14-1. The first consisting of data measured mostly from beam line 12-2 diffracted to 3.2 Å and had an overall completeness of 88.5%. The second consisting of data from both beam lines was more complete, 96.9% overall, but showed evidence of radiation damage and only diffracted to 3.25 Å. Structures were solved by molecular replacement with Phaser [67] from the CCP4 suite [68] based on the coordinates of gp120 (PDB: 3TGT), the N12-i2 Fab (PDB: 3QEG), and the CD4 mimetic peptide M48-U1 (PDB: 4JZW). Refinement was carried out with Refmac and/or Phenix [69] coupled with manual refitting and rebuilding with COOT [70].

### Structure validation and analysis

The quality of the final refined models was monitored using the program MolProbity [71]. Structural alignments were performed using the Dali server and the program lsqkab from CCP4 suite. PISA and PIC webservers were used to determine contact surfaces and residues. All illustrations were prepared with the PyMol Molecular Graphic suite (http://pymol.org) (DeLano Scientific, San Carlos, CA, USA). The models generated for each of the two data sets were largely identical. The model for the higher resolution first data set (set1) was used for PDB submission (PDB ID 6W4M) and figure generation. Data collection and refinement statistics are shown in Table 1.

### RFADCC assay

The ADCC activity of CoRBS immunoglobulins (N12-i2, 17b, X5, 412d, 48d, C11 (all generated in house [15]), and control palivizumab (MedImmune) were tested with the optimized rapid fluorometric antibody-dependent cellular cytotoxicity (RFADCC) assay [48]. Briefly, GFP-CEM-NKR-CCR5-SNAP cells sensitized with recombinant HIV-1 BaL gp120 (50 μg/ml) were used as targets and human PBMCs were utilized as effectors. Antibodies were serially diluted three-fold starting from 10 μg/ml through 0.0003 μg/ml together with positive controls mAb C11 and the negative control antibody, palivizumab, directed against respiratory syncytial virus. After 2 h of incubation, the samples were fixed and collected (at approximately 20,000 events per sample) on a Fortessa Special Order instrument (BD Biosciences) and analyzed using FlowJo software (Tree Star, Ashland, OR). ADCC activity (shown as % cytotoxicity) was defined as the percentage of GFP-CEM-NKR-CCR5-SNAP target cells that lost GFP staining but retained the CCR5-SNAP tag staining. The results represent the average of the samples tested in triplicate using a single PBMC donor.

### ADCC FACS-based assay

The infectious molecular clone NL4.3 coding for GFP, the ADA envelope with functional Nef and Vpu proteins present (ADA/WT) or deleted (ADA/Nef-Vpu-), was used as previously described [10]. In order to achieve a 20% infection rate of CEM-NKR cells, the proviral vector and a VSVG-encoding plasmid were co-transfected in 293T cells by standard calcium phosphate transfection. Concentrated virus (over a 20% sucrose cushion) was used to infect CEM-NKR cells by spin infection, as previously described [10, 52]. ADCC was measured as follows: infected CEM-NKR cells were stained with viability (AquaVivid; Thermo Fisher Scientific) and cellular (cell proliferation dye eFluor670; eBioscience) markers and used as target cells. PBMC from healthy uninfected donors were stained with another cellular marker (cell proliferation dye eFluor450; eBioscience) and used as effector cells at an effector: target ratio of 10:1 in 96-well V-bottom plates (Corning, Corning, NY). Infected cells were incubated with 0.001–5 μg/mL of CoRBS antibodies: N12-i2, 412d, X5, 48d, 17b, and Cluster A antibody C11. Plates were subsequently centrifuged for 1 min at 300*g* and incubated at 37 °C, 5% CO_2_ for 4 to 6 h before being fixed in a 2% PBS-formaldehyde solution. Samples were analyzed on an LSRII cytometer (BD Biosciences). Data analysis was performed using FlowJo vX.0.7 (Tree Star). The percentage of ADCC was calculated with the following formula: (% of GFP+ cells in Targets plus Effectors) − (% of GFP+ cells in Targets plus Effectors plus plasma)/(% of GFP+ cells in Targets) by gating on infected lived target cells. The results represent the average of the samples tested in three separate experiments utilizing two PBMC donors.

### Antibody affinity and binding to cells sensitized with gp120

To determine antibody affinity, binding to full-length single chain (FLSC) was used as a model system due to optimal exposure of the CoRBS in FLSC. Maxisorp plates were coated with 50 ng FLSC O/N before blocking and adding biotinylated serially diluted antibodies followed by streptavidin-AP and developing with two-component TMB substrate. EC50 values were then calculated using GraphPad Prism. For the investigation of Bal gp120 binding, mAbs N12-i2, 17b, X5, 412d, 48d, C11, and palivizumab were labeled using the Alexa Flour™ 647 antibody labeling kit (Thermo Fisher), as per manufacturer’s instructions. Following staining, the degree of labelling (DOL) was calculated for each sample to determine how many molecules of dye were bound to each antibody. For binding analysis, GFP-CEM-NKR-CCR5-SNAP target cells were sensitized with 50 μg/ml BaL gp120, washed and plated in V-bottom flasks at 5 × 10^4^ per well suspended in R10 [48]. Cells were incubated with AF647-stained antibodies over a range of antibody concentrations (10–0.001 μg/ml) for 20 min in the dark, followed by washing and fixing. Samples were then analyzed for AF647 staining on a LSRII flow cytometer (BD Biosciences). In order to determine the number of antibodies bound per cell (ABC), Quantum™ MESF beads (Bangs Laboratories Inc.) pre-stained with varying numbers of AF647 molecules were run alongside the cells being analyzed. A standard curve was generated using the Quickcal online tool as recommended by the kit manufacturer. The standard curve was used to calculate molecules of dye bound per cell and dividing this value by the DOL gave the number of antibodies bound per cell. The results represent the average of the samples tested in triplicate.

### Flow cytometry analysis of cell-surface staining

Cell-surface staining was performed as previously described [52]. Infected CEM-NKR cells were incubated with anti-Env antibodies (5 μg/ml) 48 h after infection. Cells were then incubated at 37 °C for 1 h followed by adding anti-human Alexa Fluor-647 (Invitrogen) secondary Abs for 20 min. The percentage of infected cells (GFP^+^) was determined by gating the live cell population on the basis of the AquaVivid viability dye staining. Samples were analyzed on an LSRII cytometer (BD Biosciences), and data analysis was performed using FlowJo vX.0.7 (Tree Star, Ashland, OR, USA). The results represent the average of the samples tested in triplicate.

### Fluorescence correlation spectroscopy (FCS) measurements

mAbs N12-i2, 17b, X5, 412d, 48d, C11, and palivizumab were labeled with Alexa 647 probe (Invitrogen mAb labelling kit) for FCS experiments. HIV-1 BaL or JRFL pseudoviruses were produced as reported previously [72]. All FCS experiments used virus preparations diluted to 10 μg/ml of p24 equivalent. gp120-to-p24 antigen ratios were typically 1:10 to 1:50. The 10 μg/ml p24 equivalent value typically corresponded to TCID50/ml values in the range of 200,000 to 650,000 (BaL) and 200,000 to 500,000 (JRFL). Dulbecco modified Eagle medium (DMEM; Gibco-BRL) was used for all FCS measurements. Pseudoviruses with 10 μg/ml p24 equivalent concentrations in 25 μl were first treated for 90 min at 37 °C with 200 μg/ml of M48U1 to expose CD4i epitopes and 100 μg/ml of nonspecific IgG1 (Calbiochem) to block non-specific binding. This was followed by addition of 5 μg/ml of Alexa Fluor 647-conjugated mAb. MAbs were allowed to interact with virions for 90 min at 37 °C. FCS measurements were performed in a confocal microscope (ISS Q2) with single-molecule detection sensitivity. The excitation source was a Fianium SC-400 super-continuum laser. A NKT super-select AOTF filter was used to select the excitation wavelength of 635 nm which was reflected by a dichroic mirror to a high-numerical-aperture (NA) water objective (60x; NA 1.2) and focused onto the solution sample. The fluorescence was collected by avalanche photodiodes through a dichroic beam splitter and a band-pass (650–720 nm; Chroma) filter, thus eliminating the scattered excitation light and collecting the fluorescence from the Alexa Fluor 647 probes in the region of interest. The data acquisition was enabled by a B&H SPC-150 card operated in a photon time-tag time-resolved (TTTR) mode. ISS VistaVision software was used to analyze the FCS data to assess the in vitro binding of mAbs to HIV-1 BaL, or JRFL pseudovirus particles. We determined the translational diffusion coefficients of Alexa 647-labeled mAbs and the corresponding complexes with virion. The percentage of total mAb (at given test concentrations) that shifts into the more slowly diffusing species which is the virion-bound fraction reflects the relative magnitude of cognate epitope exposure in the target population of virions. The FCS measurements and analyses were performed similar to previous reports [59, 72].

### Calculation of CoRBS Fab and Fc angles

Fab angles were calculated using the center of mass of gp120 and the center of mass of the CD4 d1 domain to define a vector perpendicular to the target cell membrane and the average position of the mAb CDR residues for both the heavy and light chains to define vectors for the mAbs. The Fab angle thus represents an estimate of the Fab position relative to the target cell membrane.

Fc angles were calculated using the center of mass of gp120 and the center of mass of the CD4 d1 domain to define a vector perpendicular to the target cell membrane. The Fc position was estimated by superposition of the mouse IgG (PDB ID 1IGY) onto each CoRBS mAb. Briefly, the variable part of each CORBS mAb was aligned with the variable part of Fab1 and Fab2 of 1IGY to generate two possibly Fc orientations. The average position of the C_H_2 domains of the Fc was then used to calculate a vector for the Fc position and the angle calculated from the dot product of the Fc vector and the CD4 vector and represents a tensor estimate of the position of the Fc receptor complex relative to the target cell membrane.

### Statistics

All data were analyzed using prism GraphPad Prism (version 5 for Windows, San Diego, CA, USA). Correlation analysis was via two-tailed Pearson correlation. Column analysis was analyzed via two-way ANOVA. Four asterisks represent the statistical significance of *P* < 0.0001, ***P < 0.001, **P < 0.01, and *P < 0.05.

## Supplementary information

**Additional file 1: Table S1.** Details of the N12-i2-gp120-M48U1, 412d-gp120-CD4, 48d-gp120-CD4, 17b-gp120-CD4, and X5-gp120-CD4 interfaces as calculated by the EBI PISA server (http://www.ebi.ac.uk/msd-srv/prot_int/cgi-bin/piserver). * CCR5 N-terminus, CCR5 binding site 1 of the CCR5-gp120_92BR020_-CD4d1d4 complex. ** Average of two complex copies in the asymmetric unit. *** Total for bridging sheet assembly of residues for both inner and outer domain.

**Additional file 2: Figure S1.** A 2Fo-Fc electron density map showing the two N12-i2 sulfotyrosines, 100A and 100B, bound to gp120 contoured at 1.0σ in blue and a Fo-Fc omit map of the same view, generated with tyrosine in place of sulfotyrosine, contoured at 3.0σ in green. Sulfotyrosines are as labeled. Maps generated from data set 1, 88.5% complete, are on the left and maps generated from data 2, 96.9% complete, are on the right. Positive green density from the omit maps corresponds to the placed sulfate in both sulfotyrosines.

**Additional file 3: Figure S2.** ADCC correlations. **(A)** Maximum lysis of RFADCC of BaL gp120 coated cells versus ADCC of NL43/ADA/N-U- infected. **(B)** Correlation between area under the curve (AUC) of RFADCC of BaL gp120 coated cells versus ADCC of NL43/ADA/N-U- infected. **(C)** ADCC (gp120-coated AUC) vs. antibody affinity (AUC). **(D)** ADCC (gp120-coated AUC) vs. antibody affinity (EC50). *P < 0.05 via Two-tailed Pearson correlation.

**Additional file 4: Figure S3.** Binding of antibodies to virions in solution. The binding at the single virion level was measured using fluorescence correlation spectroscopy (FCS) as described in Methods without and with the CD4 mimetic M48U1 to BaL virions (left panel) and to JRFL virions (right panel). ****P < 0.0001, ***P < 0.001 and **P < 0.01 via Two-way ANOVA. Individual data is supplied in additional files 11 and 12.

**Additional file 5: Figure S4.** Fc angle vs. ADCC lysis of ADA/N-U- infected cells infected cells. Graphs of Fc angle vs. percent lysis of ADA/N-U- infected cells at every tested antibody concentration. *P < 0.05 via Two-tailed Pearson correlation.

**Additional file 6: Figure S5.** Fc angle vs. ADCC lysis of BaL gp120 coated cells. Graphs of Fc angle vs. percent lysis of BaL gp120 coated EGFP-CEM-NKR-CCR5SNAP target cells at every tested antibody concentration.

**Additional file 7.** Individual data for Figure 5a.

**Additional file 8.** Individual data for Figure 5c.

**Additional file 9.** Individual data for Figure 6a.

**Additional file 10.** Individual data for figure 6e.

**Additional file 11.** Individual data for Figure S3 A.

**Additional file 12.** Individual data for Figure S3 B.

## Data Availability

All data generated or analyzed during this study are included in this published article, its supplementary information files, and publicly available repositories. The datasets generated are available in the Protein Data Bank (PDB) repository, http://www.rcsb.org/structure/5UWE. Individual data for Figs. 6, 7, and S3 are provided as additional files.
