# Supplementary material for: Defining rules governing recognition and Fc-mediated effector functions to the HIV-1 co-receptor binding site
Source: BMC Biol. 2020 Jul 21;18:91. doi: 10.1186/s12915-020-00819-y (PMC7374964; doi:10.1186/s12915-020-00819-y)
Supplement: Supplementary file 1 — Additional file 1: Table S1. Details of the N12-i2-gp120-M48U1, 412d-gp120-CD4, 48d-gp120-CD4, 17b-gp120-CD4, and X5-gp120-CD4 interfaces as calculated by the EBI PISA server (http://www.ebi.ac.uk/msd-srv/prot_int/cgi-bin/piserver). * CCR5 N-terminus, CCR5 binding site 1 of the CCR5-gp12092BR020-CD4d1d4 complex. ** Average of two complex copies in the asymmetric unit. *** Total for bridging sheet assembly of residues for both inner and outer domain. [file 12915_2020_819_MOESM1_ESM.docx]

**Table S1. Details of the N12-i2-gp120-M48U1, 412d-gp120-CD4, 48d-gp120-CD4, 17b-gp120-CD4, and X5-gp120-CD4 interfaces** as calculated by the EBI PISA server (<http://www.ebi.ac.uk/msd-srv/prot_int/cgi-bin/piserver>).

|  | | N12-i2 Fab-gp120_93TH057_  core_e_-M48U1 | CCR5-gp120_92BR020_-CD4 [CCR5 N-term only]*  (6MET) | 412d Fab-gp120_YU2_ core_e_-CD4  (2QAD)** | X5 Fab- gp120_JR-FL_ core_e_-CD4  (2B4C) | 48d Fab-gp120_YU2_ core_e_-CD4  (4DVR) | 17b Fab- gp120_HXBC2_ core_e_-CD4  (1GC1) |
| --- | --- | --- | --- | --- | --- | --- | --- |
| **Buried Surface Area, Å²** | **gp120 total** | **1008** | **801** | **1070** | **845** | **949** | **581** |
|  | Inner domain | 306 | 248 | 595 | 170 | 299 | 204 |
|  | Outer domain | 403 | 553 | 252 | 415 | 462 | 377 |
|  | Outer domain V3 base | 299 | 239 | 223 | 260 | 188 | 0 |
|  | Bridging sheet*** | 427 | 339 | 453 | 454 | 446 | 398 |
|  | **Heavy chain total** | **996** | - | **1108** | **786** | **634** | **471** |
|  | FWR | 162 | - | 0 | 36 | 1 | 0 |
|  | CDR H1 | 78 | - | 0 | 45 | 130 | 0 |
|  | CDR H2 | 126 | - | 285 | 260 | 388 | 203 |
|  | CDR H3 | 630 | - | 823 | 445 | 115 | 268 |
|  | TYS(1) | 213 (100A) | 174 (10) | 117 (100) | - | - | - |
|  | TYS(2) | 130 (100B) | 244 (14) | 260 (100C) | - | - | - |
|  | **Light chain total** | **34** | - | **183** | **98** | **350** | **81** |
|  | FWR | 0 | - | 50 | 0 | 31 | 0 |
|  | CDR L1 | 34 | - | 133 | 10 | 137 | 0 |
|  | CDR L2 | 0 | - | 0 | 0 | 0 | 0 |
|  | CDR L3 | 0 | - | 0 | 88 | 182 | 81 |
|  | **Heavy and light chain total** | **1030** | **998 (N-terminus)** | **1291** | **884** | **984** | **552** |

* CCR5 N-terminus,CCR5 binding site 1 of the CCR5-gp120_92BR020_-CD4d1d4 complex

** Average of two complex copies in the asymmetric unit.

*** Total for bridging sheet assembly of residues for both inner and outer domain.
