# Supplementary figures and images for: Defining rules governing recognition and Fc-mediated effector functions to the HIV-1 co-receptor binding site
Source: BMC Biol. 2020 Jul 21;18:91. doi: 10.1186/s12915-020-00819-y (PMC7374964; doi:10.1186/s12915-020-00819-y)

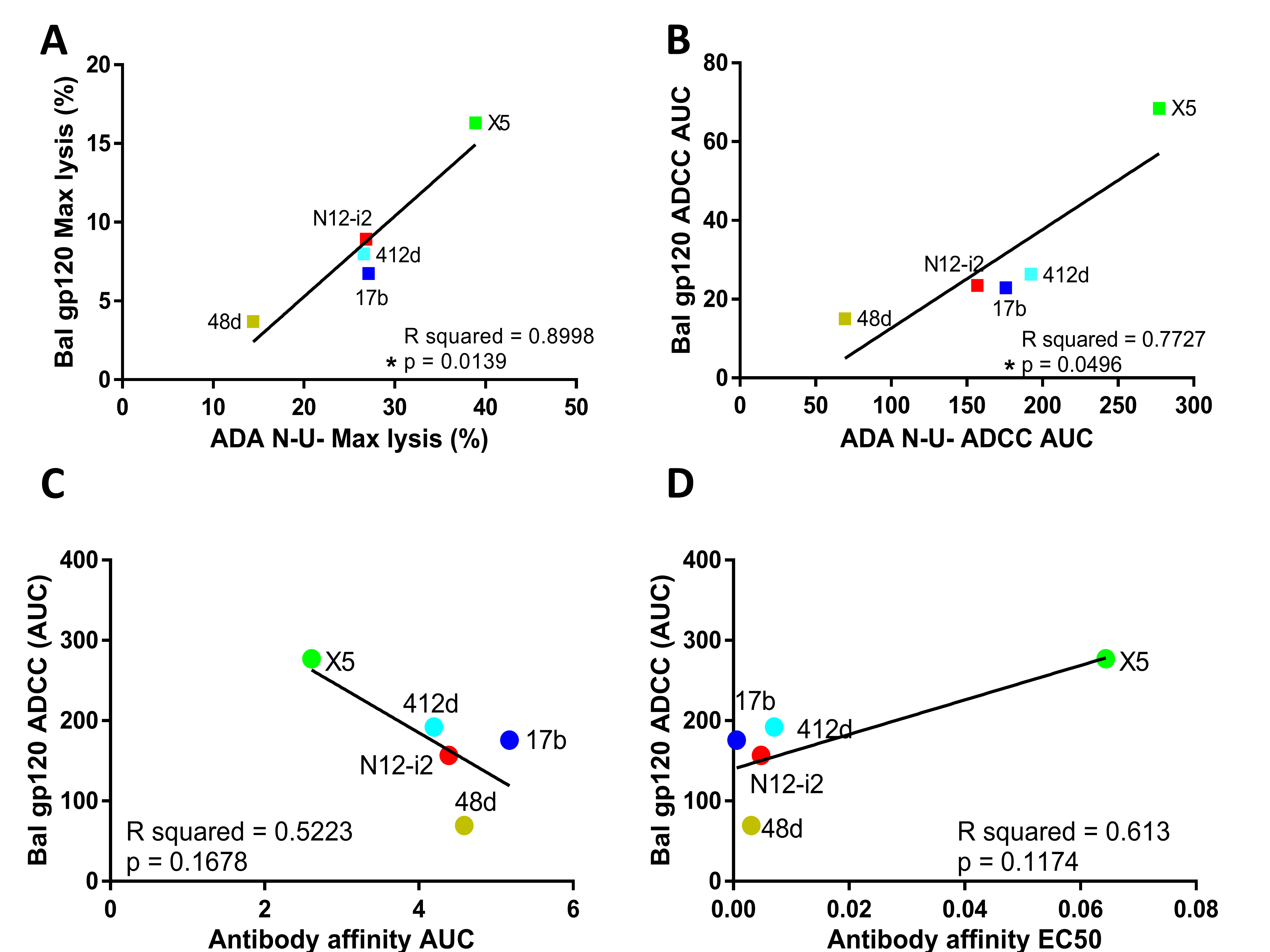

Supplement: Supplementary file 3 — Additional file 3: Figure S2. ADCC correlations. (A) Maximum lysis of RFADCC of BaL gp120 coated cells versus ADCC of NL43/ADA/N-U- infected. (B) Correlation between area under the curve (AUC) of RFADCC of BaL gp120 coated cells versus ADCC of NL43/ADA/N-U- infected. (C) ADCC (gp120-coated AUC) vs. antibody affinity (AUC). (D) ADCC (gp120-coated AUC) vs. antibody affinity (EC50). *P < 0.05 via Two-tailed Pearson correlation. [file 12915_2020_819_MOESM3_ESM.tif]

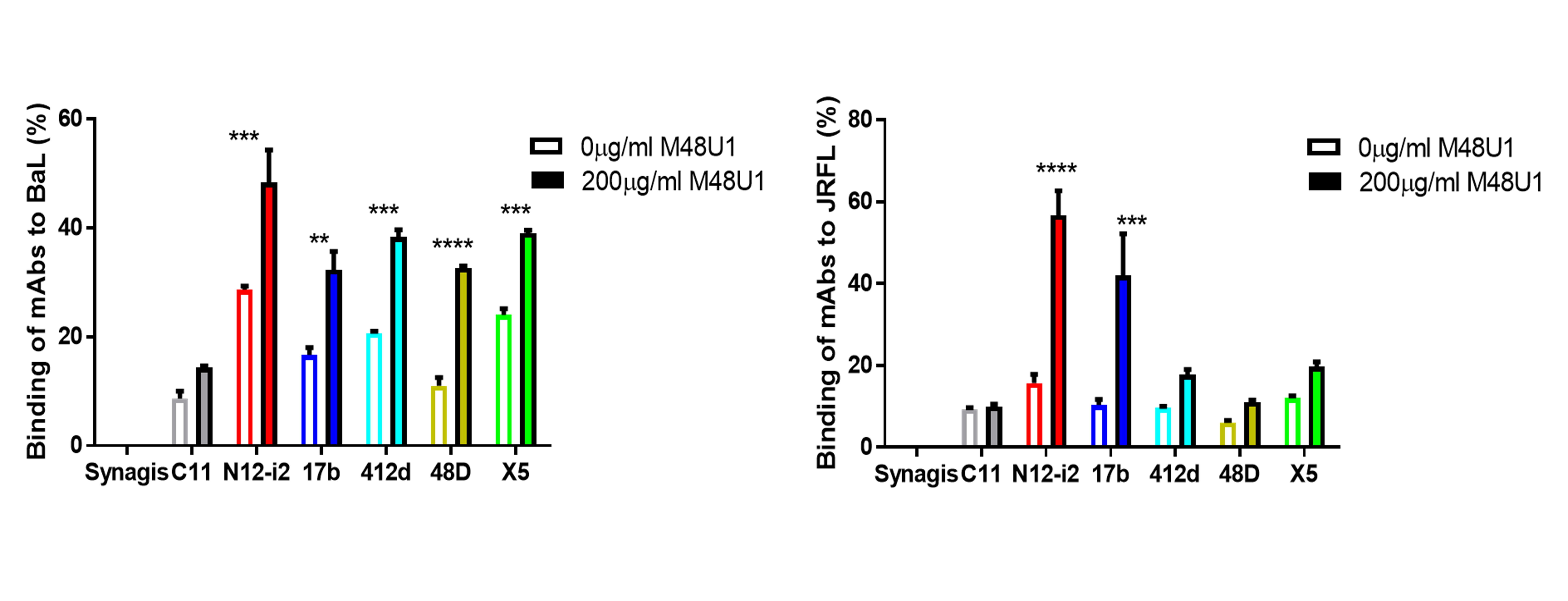

Supplement: Supplementary file 4 — Additional file 4: Figure S3. Binding of antibodies to virions in solution. The binding at the single virion level was measured using fluorescence correlation spectroscopy (FCS) as described in Methods without and with the CD4 mimetic M48U1 to BaL virions (left panel) and to JRFL virions (right panel). ****P < 0.0001, ***P < 0.001 and **P < 0.01 via Two-way ANOVA. Individual data is supplied in additional files 11 and 12. [file 12915_2020_819_MOESM4_ESM.tif]

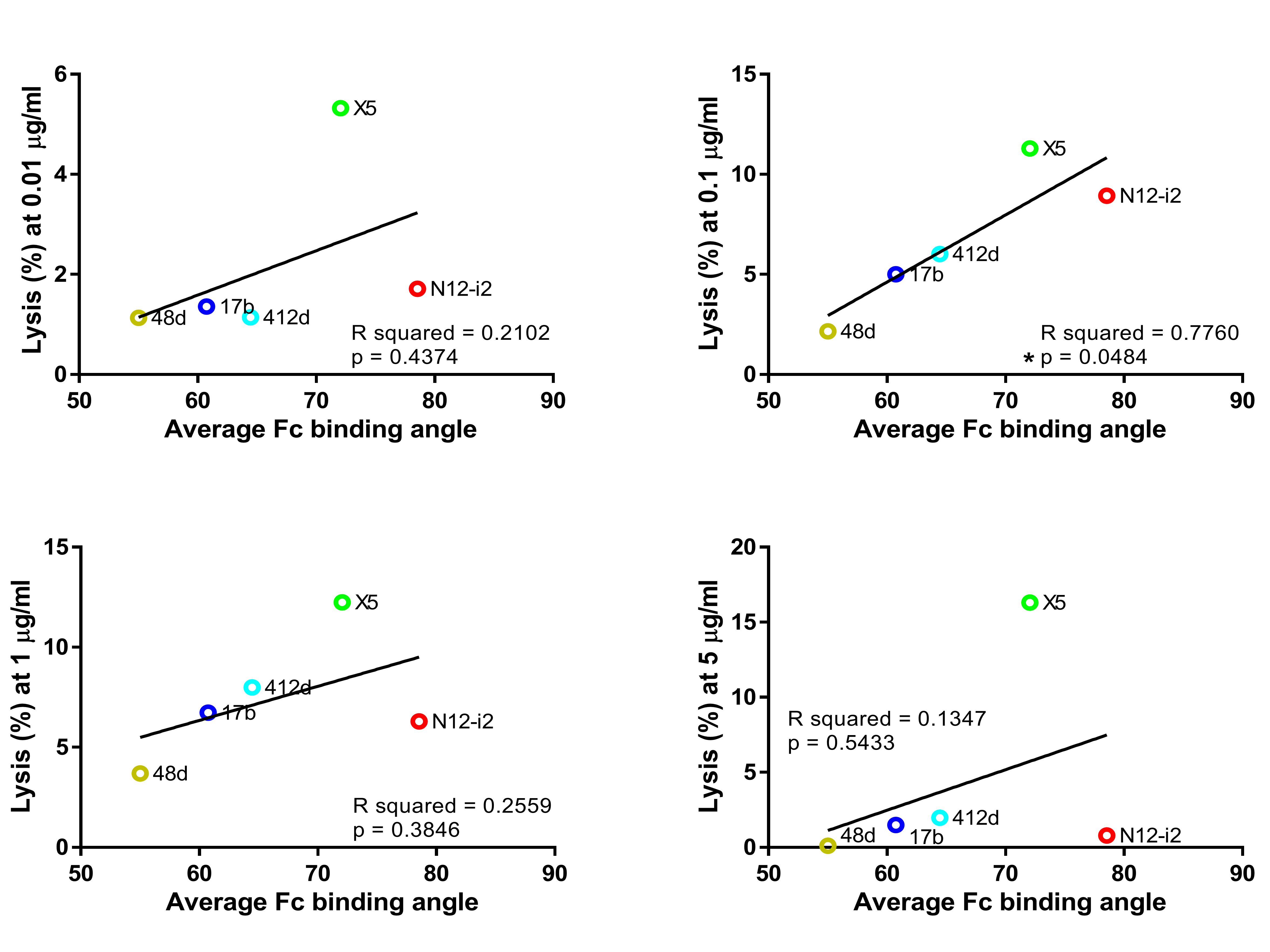

Supplement: Supplementary file 5 — Additional file 5: Figure S4. Fc angle vs. ADCC lysis of ADA/N-U- infected cells infected cells. Graphs of Fc angle vs. percent lysis of ADA/N-U- infected cells at every tested antibody concentration. *P < 0.05 via Two-tailed Pearson correlation. [file 12915_2020_819_MOESM5_ESM.tif]

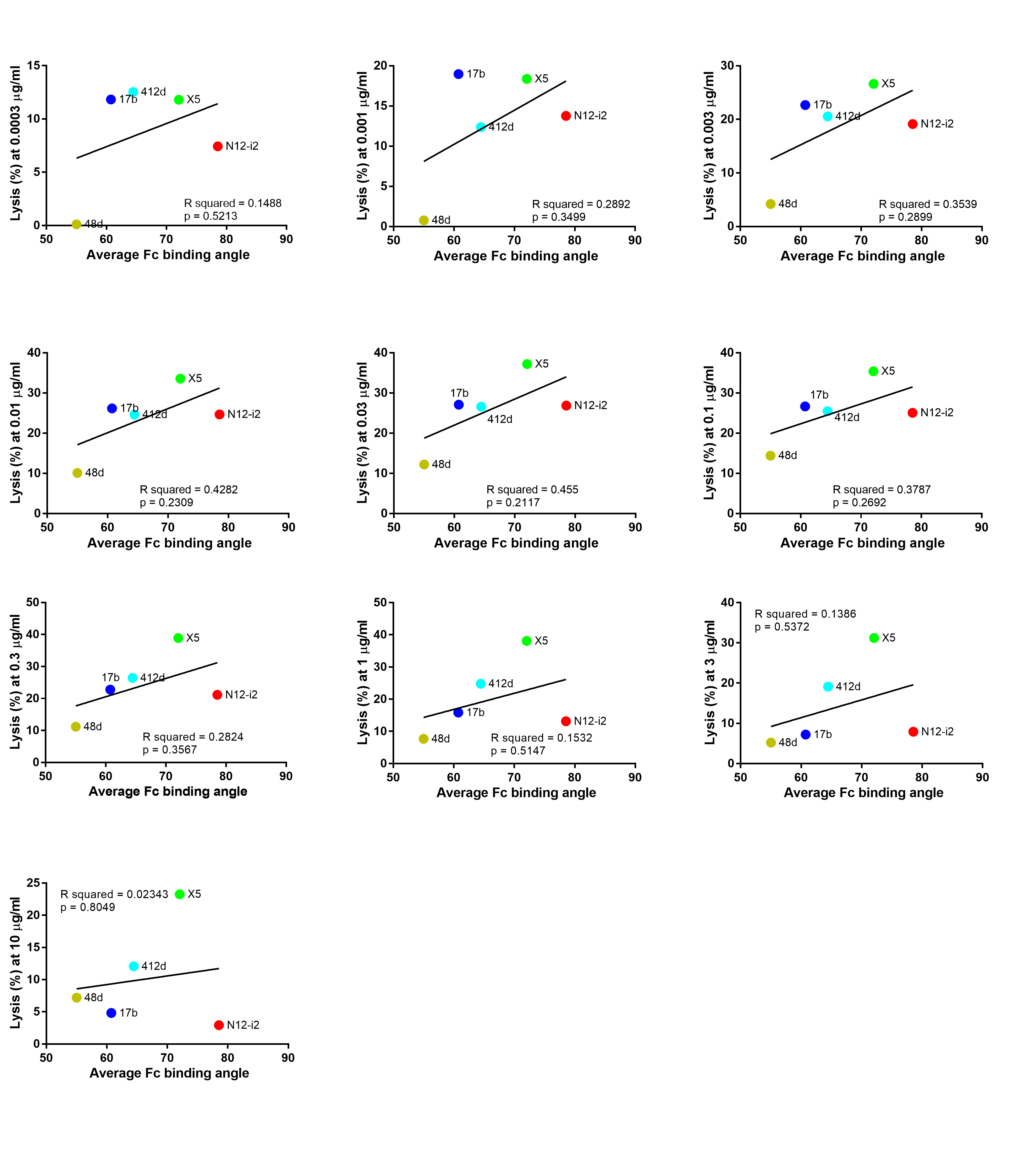

Supplement: Supplementary file 6 — Additional file 6: Figure S5. Fc angle vs. ADCC lysis of BaL gp120 coated cells. Graphs of Fc angle vs. percent lysis of BaL gp120 coated EGFP-CEM-NKR-CCR5SNAP target cells at every tested antibody concentration. [file 12915_2020_819_MOESM6_ESM.tif]
